# Attenuation of Muscle Damage, Structural Abnormalities, and Physical Activity in Respiratory and Limb Muscles following Treatment with Rucaparib in Lung Cancer Cachexia Mice

**DOI:** 10.3390/cancers14122894

**Published:** 2022-06-11

**Authors:** Maria Pérez-Peiró, Xavier Duran, José Yélamos, Esther Barreiro

**Affiliations:** 1 Muscle Wasting and Cachexia in Chronic Respiratory Diseases and Lung Cancer Research Group, Pulmonology Department, Department of Medicine and Life Sciences (MELIS), Hospital del Mar, Medical Research Institute (IMIM), Parc de Salut Mar, Universitat Pompeu Fabra (UPF), Barcelona Biomedical Research Park (PRBB), 08003 Barcelona, Spain; mperez4@imim.es; 2 Centro de Investigación en Red de Enfermedades Respiratorias (CIBERES), Instituto de Salud Carlos III (ISCIII), 08003 Barcelona, Spain; 3 Scientific, Statistics and Technical Department, Hospital del Mar, Medical Research Institute (IMIM), Parc de Salut Mar, 08003 Barcelona, Spain; xduran@imim.es; 4 Cancer Research Program, Hospital del Mar, Medical Research Institute (IMIM), 08003 Barcelona, Spain; jyelamos@imim.es

**Keywords:** respiratory and limb muscles, lung-cancer-induced cachexia, PARP inhibitor rucaparib, muscle structural abnormalities, muscle damage, physical activity, proteolytic and autophagy markers

## Abstract

**Simple Summary:**

Muscle wasting and cachexia are common in patients with cancer. Several mechanisms underlie muscle physiological and structural alterations in cancer-induced cachexia. Poly (ADPribose) polymerases (PARPs) are involved in muscle metabolism and in cancer. Selective inhibitors of PARP activity improve muscle function and structure. This study sought to investigate whether rucaparib (PARP inhibitor) may attenuate muscle damage in a mouse model of lung-cancer-induced cachexia. Rucaparib was administered to cancer-cachectic mice. Physiological and biological parameters were determined in the respiratory and limb muscles of the animals. In cancer cachexia mice compared to non-cachexia controls, body weight and body weight gain, muscle weight, limb strength, physical activity, and muscle fiber size significantly declined, while levels of PARP activity, plasma troponin I, muscle damage, and proteolytic and autophagy markers increased. Treatment with rucaparib elicited a significant improvement in body weight gain, tumor size and weight, physical activity, muscle damage, troponin I, and proteolytic and autophagy levels.

**Abstract:**

Overactivation of poly (ADPribose) polymerases (PARPs) is involved in cancer-induced cachexia. We hypothesized that the PARP inhibitor rucaparib may improve muscle mass and reduce damage in cancer cachexia mice. In mouse diaphragm and gastrocnemius (LP07 lung adenocarcinoma) treated with PARP inhibitor (rucaparib,150 mg/kg body weight/24 h for 20 days) and in non-tumor control animals, body, muscle, and tumor weights; tumor area; limb muscle strength; physical activity; muscle structural abnormalities, damage, and phenotype; PARP activity; and proteolytic and autophagy markers were quantified. In cancer cachexia mice compared to non-cachexia controls, body weight and body weight gain, muscle weight, limb strength, physical activity, and muscle fiber size significantly declined, while levels of PARP activity, plasma troponin I, muscle damage, and proteolytic and autophagy markers increased. Treatment with the PARP inhibitor rucaparib elicited a significant improvement in body weight gain, tumor size and weight, physical activity, muscle damage, troponin I, and proteolytic and autophagy levels. PARP pharmacological inhibition did not exert any significant improvements in muscle weight, fiber size, or limb muscle strength. Treatment with rucaparib, however, improved muscle damage and structural abnormalities and physical activity in cancer cachexia mice. These findings suggest that rucaparib exerts its beneficial effects on cancer cachexia performance through the restoration of muscle structure.

## 1. Introduction

Oncologic cachexia is a major systemic manifestation in many cancer types [1,2,3,4]. Muscle wasting is probably the most characteristic feature of cancer cachexia in patients and animal models [5,6,7,8,9]. Muscle mass loss usually entails the impairment of muscle function at the cellular and myofiber levels. Skeletal muscle weakness leads to poor exercise tolerance, which negatively impacts the patients’ daily activity. Reduced physical activity is a hallmark of patients with oncologic cachexia, resulting in reduced quality of life [6,10,11]. On the other hand, poor prognosis is also another characteristic feature of cancer cachexia in clinical settings. Survival is a relevant outcome as death occurs significantly earlier in patients with advanced muscle wasting and cachexia [12,13,14].

In the etiology of cancer cachexia, several clinical factors and cellular mediators are responsible for the deterioration of muscle fiber size and function [3,5,6,11,15]. The hypermetabolic activity of the tumor, reduced physical activity, cancer therapies, and increased oxidative stress and inflammation are counted among the most relevant factors and mechanisms involved in the process of oncologic cachexia. Other mechanisms such as increased proteolysis and autophagy may also mediate the process of muscle wasting in cancer cachexia and other diseases [7,8,9,16,17,18,19,20,21]. Furthermore, muscle damage and structural abnormalities were also shown to be increased in the skeletal muscles both in patients with cancer cachexia [22,23] and in animal models of cancer [7,16,23]. 

ADP-ribosyl transferases are a family of enzymes that transfer one or several ADP-ribose units from donor β-nicotinamide adenine dinucleotide (NAD^+^) molecules to acceptor target proteins, thus inducing protein mono-ADP-ribosylation (MARylation) or poly-ADP-ribosylation (PARylation) [24]. PARP-1 (80–85%) and PARP-2 (5–10%) are the most important contributors to PARylation in tissues [25,26], extensively expressed in the cells of almost all human tissues, including the nervous system, adipose tissue, hormone glands, liver, and skeletal muscle [17,27,28,29]. PARP-1 and PARP-2 enzymes are involved in different biological functions ranging from maintenance of genomic stability, RNA transcription, DNA repair, protein translation and degradation to cell death, metabolism and mitochondrial function, and cell differentiation and aging [30,31,32,33,34,35]. 

Hyperactivation of PARP-1 and/or PARP-2 may underlie several diseases such as acute lung and renal injuries [35,36,37], cardiovascular diseases [38], and sepsis [39]. Specific genetic deletion of PARP-1 and PARP-2 in lung cachectic mice prevented the loss of muscle mass and function through several mechanisms such as attenuation of muscle proteolysis, oxidative stress, and epigenetics in cancer-cachectic mice [17,29]. Pharmacological inhibition of PARP1/2 activity and genetic deletion attenuated muscle damage within the myofibers and also reduced systemic troponin I levels in experimental models [40,41]. 

In clinical settings, PARP-1/2 inhibitors are currently being used for the treatment of certain cancer types, namely ovarian and breast cancer [42,43,44,45]. Olaparib, rucaparib, talazoparib, and niraparib have been approved for the treatment of some tumors bearing homologous recombination DNA repair deficiencies in North America and Europe, and many clinical trials are also underway to try to extend the number of patients who can benefit from PARP-based therapies [42,45,46,47]. Whether inhibition of PARP-1/2 activity may partly revert the loss of muscle mass and damage in oncologic cachexia remains to be fully understood. 

Hence, we hypothesized that the PARP-1/2 inhibitor rucaparib may improve muscle mass and reduce damage in lung cancer cachectic mice. To address this issue, we used a mouse model of lung cancer that induces cachexia by inoculation of the LP07 adenocarcinoma cell line. Mice were treated with rucaparib for 20 consecutive days, and the following parameters were analyzed: (1) total body, muscle, tumor weights, and tumor area; (2) limb muscle strength, and physical activity; (3) muscle structural abnormalities and damage; (4) muscle phenotype; (5) PARP activity; and (6) markers of muscle proteolysis and autophagy. A group of non-treated cachectic mice were also analyzed, as well as non-cachexia control animals. 

## 2. Materials and Methods

### 2.1. Experimental Model and Design

The in vivo experiments were carried out in the animal facilities at Barcelona Biomedical Research Park (PRBB) under specific-pathogen-free conditions. The following ethical standards on animal experimentation, i.e., EU 2010/63 CEE, *Real Decreto* 53/2013 BOE 34, Spain, and the Helsinki convention for the use and care of animals were followed. The Animal Research Committee (Animal welfare department, Catalonia, EBP-17-0005) granted ethical approval for the project. 

A P07 lung tumor that developed spontaneously in the lung of a BALB/c mouse was isolated to generate the LP07 murine cell line, which was used in the current study [7,8,48,49,50]. The LP07 cell line was graciously provided by Dr. Urtreger, Dr. Diament, and Dr. Bal de Kier Joffé (Research Area Institute of Oncology “Angel H. Roffo”, Buenos Aires, Argentina). LP07 cells were kept at 37 °C in a humidified, 5% CO_2_–air atmosphere in minimal essential media (MEM, ref: L0415-500, Biowest, Nuaille, France) supplemented with 10% fetal bovine serum (FBS) (Thermo Fisher Scientific Inc., Waltham, MA, USA) and 1% of penicillin/streptomycin/fungizone solution: 10,000 U/mL penicillin, 10,000 µg/mL streptomycin, and 25 µg/mL fungizone (Thermo Fisher Scientific Inc.). Subcultures were carried out using trypsin–ethylenediaminetetraacetic acid (EDTA) 1X in phosphate-buffered saline (PBS, Thermo Fisher Scientific Inc.). Cells were expanded for three weeks in order to reach the required amounts of cells to be inoculated to the mice. Subsequently, 4 × 10^5^ LP07 cells, resuspended in 0.2 mL MEM, were injected subcutaneously in the left flank of the mice on day 0 [7,8,17,29,50].

**Figure 1:**
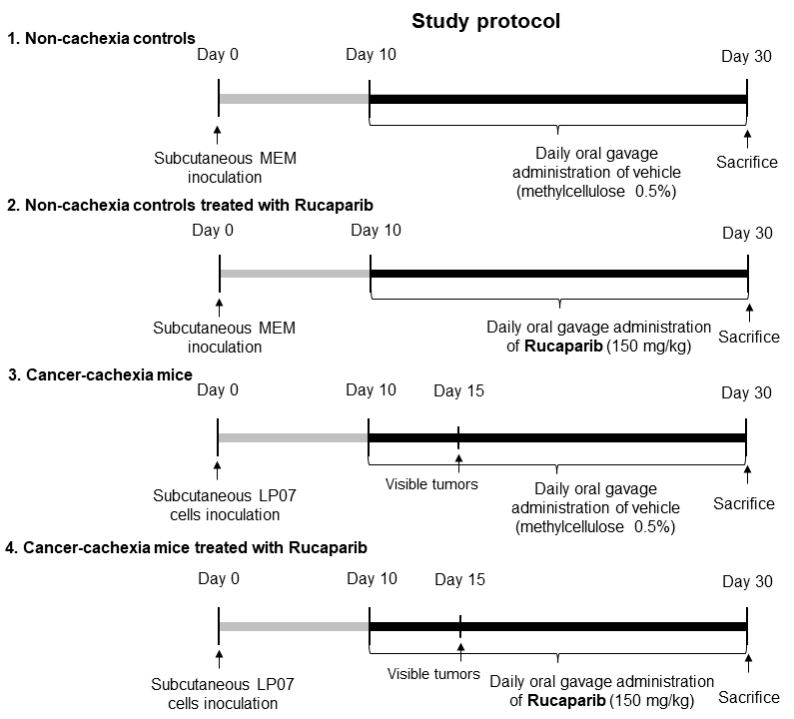
Schematic time-line representation of the study protocol in the four studied groups.

Rucaparib was kindly provided by Clovis Oncology (San Francisco, CA, USA) for the purpose of this study. Rucaparib (150 mg/kg in 0.5% methylcellulose) treatment started on day 10 up until day 30 (Figure 1). Animals were treated daily (gavage procedures using a specific 20 G reusable feeding needle (Fine science tools Inc., Foster City, CA, USA) using fresh-daily rucaparib solution. Non-treated mice (sham controls) were administered 0.5% methylcellulose dissolved in distilled water (gavage) daily from day 10 up until day 30. All the mice were sacrificed on day 30.

Forty BALB/c ten-week-old female mice (*n* = 10/group) were obtained from Harlan Interfauna Ibérica SL (Barcelona, Spain). Animals were divided into four different groups: (1) non-cachexia control group, treated daily with 0.5% methylcellulose, 0.2 mL MEM inoculated in the left flank; (2) non-cachexia control group treated daily with 150 mg/kg rucaparib, 0.2 mL MEM inoculated in the left flank; (3) lung cancer cachexia group, treated daily with 0.5% methylcellulose, inoculation of LP07 cells resuspended in 0.2 mL MEM in the left flank; and (4) lung cancer cachexia group treated with 150 mg/kg/24 h rucaparib, inoculation of LP07 cells resuspended in 0.2 mL MEM in the left flank. In the cancer-cachectic mice, the tumors were visible from day 14 thereafter up to the end of the study protocol (30 days).

### 2.2. Sacrifice and Sample Collection

All the animals were sacrificed on day 30 using an intraperitoneal injection of 0.1 mL sodium pentobarbital (60 mg/kg) to induce anesthetic depth. Confirmation of death in euthanized animals was verified by evaluating the pedal and blink reflexes.

Diaphragm and gastrocnemius muscles and tumors were obtained from all the mice. In both types of muscle samples, a fragment of the muscle specimens was immediately frozen in liquid nitrogen and was preserved at −80 °C for further molecular analysis. Furthermore, the remaining specimens of diaphragm and gastrocnemius were immersed in an alcohol–formol bath to be thereafter embedded in paraffin as previously described [7,18,29,50]. Additionally, blood samples were obtained through puncture of the saphenous vein on day 30. Blood was collected with a capillary blood collector (Microvette® 200 LH, Sarstedt Inc., Newton, NC, USA) and tubes were centrifuged at 3000× *g* at 4 °C for 15 min. The yielded plasma was aliquoted and kept at −80 °C until further use.

### 2.3. In Vivo Measurements in the Mice

During the study period, food and water were supplied ad libitum to all the animals. Body weight and food intake were measured every 24 h. Tumor area was determined using a caliper and the formula: (L × W^2^)/2, where L is tumor length and W is tumor width. Tumor weight was determined using a scale at the end of the study. Physical activity was also identified at the end of the study protocol using a specific equipment (Oxyletpro system, Panlab, S.L.U, Barcelona, Spain). Four-limb grip strength measurements were measured using a specific grip meter (Bioseb, Vitrolles Cedex, France) on days 0 and 30 [7,18,29,50]. Grip strength measurements were normalized to body weight in all the mice [51,52].

### 2.4. Biological Analysis

#### 2.4.1. PARP activity

Gastrocnemius and diaphragm paraffin-embedded sections (three micrometers) were deparaffinized with xylene and rehydrated through a graded ethanol series. Antigen retrieval was performed with 1 mM ethylenediaminetetraacetic acid (EDTA) buffer and 0.05% of Tween20 (pH 8.0) at 95 °C for 40 min. After cooling for 30 min, endogenous peroxidase activity was blocked with 6% of H_2_O_2_. Sections were then rinsed in PBS and were subsequently incubated with mouse IgG blocking reagent (MOM) (Vector Laboratories, Newark, CA, USA) at room temperature for 30 min. Slides were incubated at room temperature with pADPr polymer antibody (Anti-PAR, Santa Cruz Biotechnology, Dallas, TX, USA) for 40 min. Sections were again rinsed with PBS three additional times (Sigma-Aldrich, St. Louis, MO, USA). Slides were incubated with horseradish peroxidase (HRP) polymer-antiMouse/Rabbit IgG (Neobiotech, Seoul, Korea) at room temperature for 30 min. After three washes with PBS, 3,3′-diaminobenzidine (DAB) solution (Neobiotech, Seoul, Korea) was applied until the appropriate color (brown) was reached. Samples were rinsed under tap water for 10 min. Slides were counter stained with hematoxylin. Sections were dehydrated with an alcohol–xylene battery and were mounted in dibutylphthalate polystyrene xylene (DPX) media [17,53]. Negative control samples (PBS-only incubations) were run in each assay. Images of the stained sections were captured using a light microscope (×40 objective, Olympus, Series BX50F3, Olympus Optical Co., Hamburg, Germany). Positive stained nuclei for pADPr polymers were counted using ImageJ software (National Institute of Health, available at http://rsb.info.nih.gov/ij/, accessed on 1 February 2022).

#### 2.4.2. Enzyme-Linked Immunosorbent Assay (ELISA) Plasma Skeletal Muscle Troponin I Levels

Skeletal muscle troponin I levels were quantified in plasma samples of the four study groups using a specific sandwich ELISA kit (Elabscience Biotechnology Inc., Houston, TX, USA) following the manufacturer’s instructions. A standard curve was always run with each assay. Briefly, 100 μL plasma samples (1:3 dilution) were poured onto the wells. Both standards and samples were loaded in duplicates onto the pre-coated ELISA plate wells. Samples were incubated at 37 °C for 90 min. Immediately afterward, the samples were incubated with biotinylated detection antibody working solution at 37 °C for one hour. After three washes, samples were incubated with HRP-conjugated working solution at 37 °C for 30 min. Followed by five more washes, substrate reagent was incubated at 37 °C for 15 min. The stop solution stopped the enzyme reaction. Optical densities (ODs) were obtained in a micro-plate reader (Infinite M Nano, Tecan Group Ltd., Zürich, Switzerland) using a wavelength of 450 nm. Intra-assay coefficients of variation for this assay were lower than 10%. As all the samples were analyzed on the same day; no inter-assay coefficients of variation could be calculated.

#### 2.4.3. Muscle Structure Abnormalities

On 3 μm paraffin-embedded sections of the gastrocnemius and diaphragm muscles, alterations in muscle structure and phenotype were assessed following previous methodologies [17,18,23,54,55]. Hematoxylin–eosin was used to stain the muscle cross-sections. Subsequently, images were captured using a light microscope (×40 objective, Olympus, Series BX50F3, Olympus Optical Co., Hamburg, Germany). Normal and abnormal muscle fractions were identified on the study images that were previously superimposed on a 63-square grid composed of a 7 × 9 rectangular pattern. The following items were analyzed on each square of the grids: (1) normal muscle, (2) internal nucleus, (3) inflammatory cell, (4) lipofuscin, (5) abnormal viable fiber, (6) necrotic fiber, (7) blood vessel, and (0) no count.

The percentage of all the points that fell between categories 2 and 6 were used to calculate the abnormal muscle fractions for the studied muscles. Internal nuclei, inflammatory cells, and necrotic cells were defined as the percentage of points that fell into categories 2, 3, and 6, respectively, relative to the total counted points Categories 0 and 7 were not considered in the counting.

#### 2.4.4. Muscle Fiber Typing and Morphometry

Slow- and fast-twitch muscle fibers were identified using specific antibodies using immunohistochemistry in the diaphragm and gastrocnemius sections of all the mice. MyHC-I and -II isoforms were identified using anti-MyHC-I and anti-MyHC-II antibodies (Abcam, Cambridge, UK), following previously published methodologies [7,8,9,17,50]. Muscle paraffin-embedded sections were deparaffinized with xylene and rehydrated through a graded ethanol series. Antigen retrieval was performed with 1 mM EDTA buffer with 0.05% of Tween20 (pH 8.0) at 95 °C for 40 min. Endogenous peroxidase activity was blocked with 6% of H_2_O_2_ after cooling down the samples for 30 min. The sections were rinsed with PBS and were subsequently incubated with MOM at room temperature for 30 min. Slides were incubated with the corresponding primary antibodies at 4 °C overnight. The sections were rinsed with PBS three times and were immediately incubated with HRP polymer-antiMouse/Rabbit IgG (Neobiotech, Seoul, Korea) at room temperature for 30 min. Following three washes with PBS, DAB solution was applied until the appropriate color (brown) was reached, and samples were rinsed with tap water. Sections were counter stained using hematoxylin. Sections were dehydrated with an alcohol–xylene battery and mounted in DPX media. Negative control samples (blocking solution incubations) were run in each assay. Images of the stained sections were captured using a light microscope (×20 objective, Olympus, Series BX50F3, Olympus Optical Co., Hamburg, Germany). Cross-sectional area (CSA), mean least diameter, and the proportions of slow- and fast- twitch muscle fibers were analyzed using ImageJ software (National Institute of Health, available at http://rsb.info.nih.gov/ij/, accessed on 1 February 2022). In each muscle cross-section, at least 100 fibers were measured and counted, separately, from all the study groups of mice.

#### 2.4.5. Immunoblotting

Frozen muscle samples from the diaphragm and gastrocnemius of all experimental mice groups were homogenized using a specific lysis buffer, as previously described [7,9,17]. The Bradford method was applied to quantify protein levels of muscle homogenates as previously described [7,8,9,17,18,19,50].

Protein samples (ranging from 5 to 20 μg, according to antigen and antibody) were diluted with an equal volume of 2X Laemmli buffer (Bio-Rad Laboratories, Inc., Hercules, CA, USA) and 10% of 2-mercaptoethanol (Bio-Rad Laboratories). Subsequently, samples were boiled for 5 min at 95 °C and were separated using electrophoresis. Proteins were transferred onto polyvinylidene difluoride (PVDF) membranes, blocked with bovine serum albumin (BSA) or with 5% nonfat milk, and incubated with primary antibodies overnight at 4 °C. The following primary antibodies were used to analyze the protein content of the target biomarkers: muscle ring finger (MURF)-1 (anti-MURF-1 antibody, Santa Cruz Biotechnology), ubiquitin-ligase atrogin-1 (anti-atrogin-1 antibody, Acris), 20S proteasome subunit C8 (anti-C8 antibody, Biomol, Plymouth Meeting, PA, USA), total ubiquitinated proteins (anti-ubiquitinated proteins antibody, Boston Biochem, Cambridge, MA, USA), nucleoporin p-62 (anti-p62/SQSTM1 antibody, Sigma-Aldrich, St. Louis, MO, USA), beclin-1 (anti-beclin-1 antibody, Santa Cruz Biotechnology), light chain LC3B (anti-LC3B antibody, Cell Signaling, Boston, MA, USA), and glyceraldehyde-3-phosphate dehydrogenase (GAPDH, anti-GAPDH antibody, Santa Cruz Biotechnology). Positive controls were used to assess the specific band in ubiquitin-ligase atrogin-1 (MAFBx 239T lysate, Santa Cruz Biotechnology) and MuRF-1 (MuRF-1 239T lysate, Santa Cruz Biotechnology). Antigens from all the samples were detected using HRP-conjugated secondary antibodies (Jackson ImmunoResearch Inc., West Grove, PA, USA) and a chemiluminescence kit (Thermo Scientific, Rockford, IL, USA). PVDF membranes from the different groups were scanned at the same time under identical exposure conditions using an Alliance Q9 Advanced imager (Uvitec Cambridge, UK). The optical densities of specific bands were quantified using the ImageJ software (National Institute of Health, available at http://rsb.info.nih.gov/ij/, accessed on 1 February 2022). Membranes were stripped of primary and secondary antibodies through incubation with stripping solution (25 nM glycine, pH 2.0, and 1% sodium dodecyl sulfate, SDS) for 30 min according to previously published methodologies [7,8,9,17,18,19,50] to detect the protein loading control GAPDH for each of the markers. The optical densities obtained from each study marker were normalized to those of the loading control (GAPDH).

### 2.5. Statistical Analysis

Data are presented as mean values (standard deviation). The Shapiro–Wilk test was conducted to explore the normality of the study variables. The muscle damage marker troponin I was chosen as the outcome variable in the study. Statistical power was found to be almost 80% (77.68%) for the four study groups, in which 10 animals were analyzed in each group. Two levels of comparisons were established for all the study variables: (1) comparisons between any experimental group and non-cachexia control group and (2) comparisons between the lung cancer cachexia group and the lung cancer cachexia group treated with rucaparib. Unpaired Student’s *t*-test was used to explore differences between two study groups, while two-way analysis of variance (ANOVA) was used to analyze the following effects: lung cancer cachexia, treatment with rucaparib, and the interaction between these two factors for all the study variables. Potential associations between two variables were assessed using the Pearson’s correlation coefficient. Moreover, potential differences between two specific groups were analyzed using contrast of marginal linear predictions. Statistical analyses were performed using STATA (software for Statistics and Data Science) software (StataCorp LLC, College Station, TX, USA).

## 3. Results

### 3.1. Rucaparib Influenced Body Weight and Physical Activity in Cancer Cachexia

Compared to non-cachexia controls, the following parameters were significantly reduced in tumor-bearing mice: body weight and body weight gain, muscle weight, limb strength, and physical activity (Table 1). Treatment with the PARP-1/2 inhibitor rucaparib elicited a significant improvement in body weight gain, tumor size and weight, and locomotor movements compared to non-treated cachexia mice (Table 1). No significant differences were seen in body weight gain, tumor size or weight, muscle weight, limb strength, or locomotor activity between the two non-cachexia controls (Table 1).

### 3.2. Rucaparib Attenuated PARP Activity in Respiratory and Limb Muscles in Cancer Cachexia

**Figure 2:**
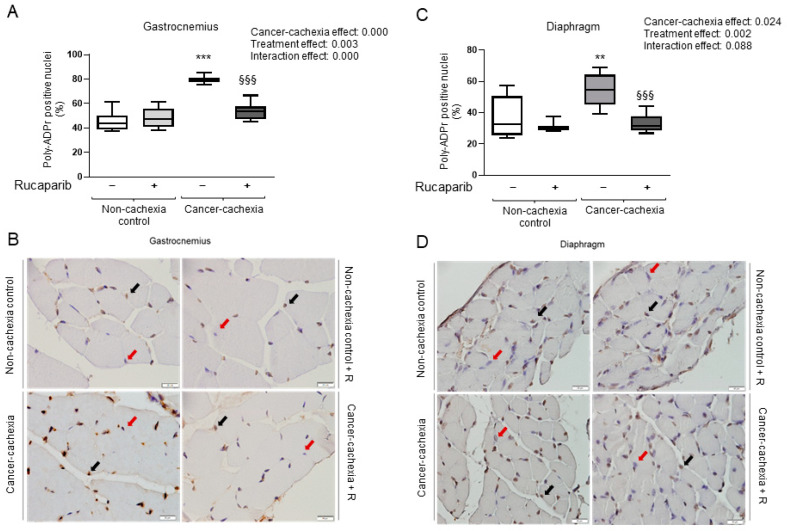
Box plot representation of PARP activity measured as the percentage of positively stained nuclei for poly-ADPr in the gastrocnemius (**A**) and diaphragm (**C**). Representative images of nuclei positively stained for poly-ADPr (black arrows point toward brown nuclei) and not stained (red arrows point toward blue nuclei, hematoxylin counterstaining) in the gastrocnemius (**B**) and diaphragm (**D**) of the different experimental groups of mice. Abbreviations: poly-ADPr, poly-ADP ribosylation; −, untreated with rucaparib; +, treated with rucaparib. Statistical significance: ** *p* ≤ 0.01, and *** *p* ≤ 0.001 between the untreated non-cachexia controls and the non-treated cancer cachexia mice; ^§§§^ *p* ≤ 0.001 between the cancer cachexia mice treated with rucaparib and the non-treated cancer cachexia mice. Two-way ANOVA significance (*p* value) of cancer cachexia and treatment and interaction effects are also shown for each variable.

The diaphragm and gastrocnemius of cancer cachexia mice showed a significant rise in PARP activity compared to non-cachexia controls (Figure 2). Treatment with rucaparib, however, elicited a significant decline in PARP activity in both muscles in the cancer cachexia mice compared to the non-treated animals (Figure 2).

### 3.3. Effects of Rucaparib on Muscle Damage

**Figure 3:**
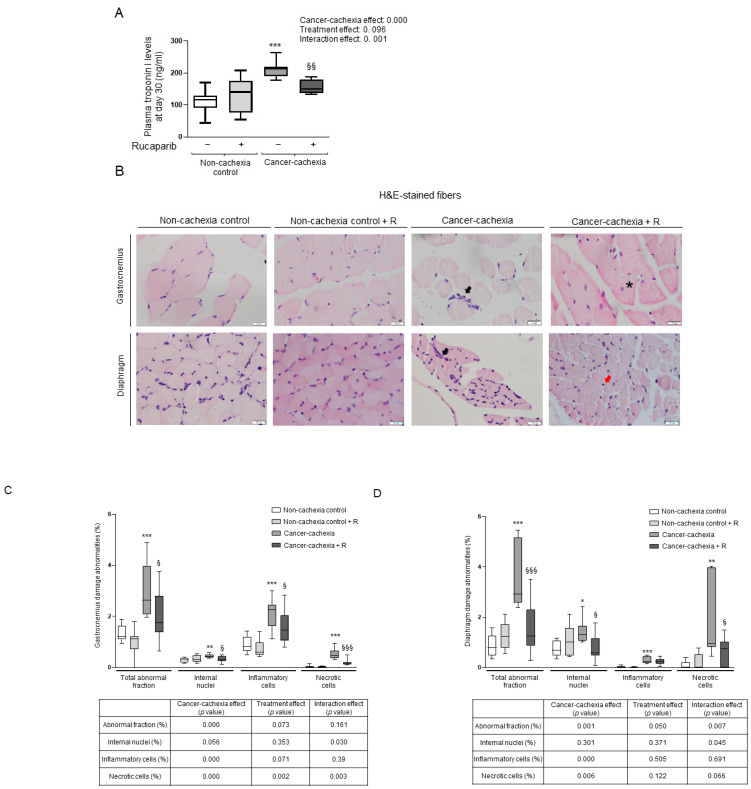
Box plot representation of troponin I plasma levels (ng/mL) at day 30 in all the studied groups (**A**). Representative images of muscle morphology within the gastrocnemius (top panel) and diaphragm (bottom panel) (**B**). An example of a necrotic cell (black arrow), internal nuclei (asterisk), and an inflammatory cell (red arrow) are indicated in the panels. Box plot representation of the percentage of total abnormal fraction, internal nuclei, inflammatory cells, and necrotic cells in the gastrocnemius (**C**) and diaphragm (**D**) of all experimental groups. Definition of abbreviations: R, rucaparib. H&E, hematoxylin and eosin. Statistical significance: * *p* ≤ 0.05, ** *p* ≤ 0.01, and *** *p* ≤ 0.001 between the untreated non-cachexia controls and the non-treated cancer cachexia mice; ^§^ *p* ≤ 0.05, ^§§^ *p* ≤ 0.01, and ^§§§^ *p* ≤ 0.001 between the cancer cachexia mice treated with rucaparib and the non-treated cancer cachexia mice. Two-way ANOVA significance (*p* value) of cancer cachexia and treatment and interaction effects are also shown for each variable.

Plasma levels of the muscle damage marker troponin I were significantly increased in cancer cachexia mice compared to non-cachexia controls, while treatment with rucaparib significantly attenuated such an increase in the treated cachectic animals (Figure 3A). Inverse correlations were found between grip strength and troponin I levels in the non-treated and treated cachectic mice (r = −0.892 and *p* = 0.01 and r = −0.541 and *p* = 0.037). The proportions of abnormal muscle, internal nuclei, and inflammatory and necrotic cells significantly increased in both the diaphragm and the gastrocnemius of cancer cachexia mice compared to non-cachectic controls, and rucaparib elicited a significant decline in those parameters, except for inflammatory cell counts in the diaphragm, in the treated animals (Figure 3B,D, respectively).

### 3.4. Muscle Phenotype and Morphometry

**Figure 4:**
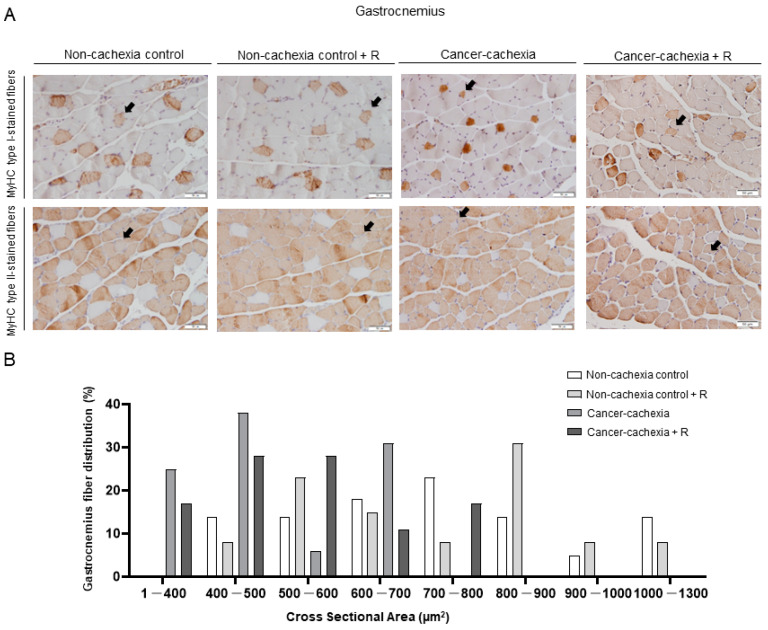
Representative images of gastrocnemius muscle fibers (**A**). Myofibers with anti-MyHC type I antibody staining (brown color) are shown in the top panel and anti-MyHC type II antibody staining in the middle panel. Hybrid fibers (arrows) are seen in both panels. Schematic representation of the distribution of the CSA of the muscle fibers in the gastrocnemius muscle (**B**). Abbreviations: R, rucaparib; MyHC, myosin heavy chain; CSA, cross-sectional area.

**Figure 5:**
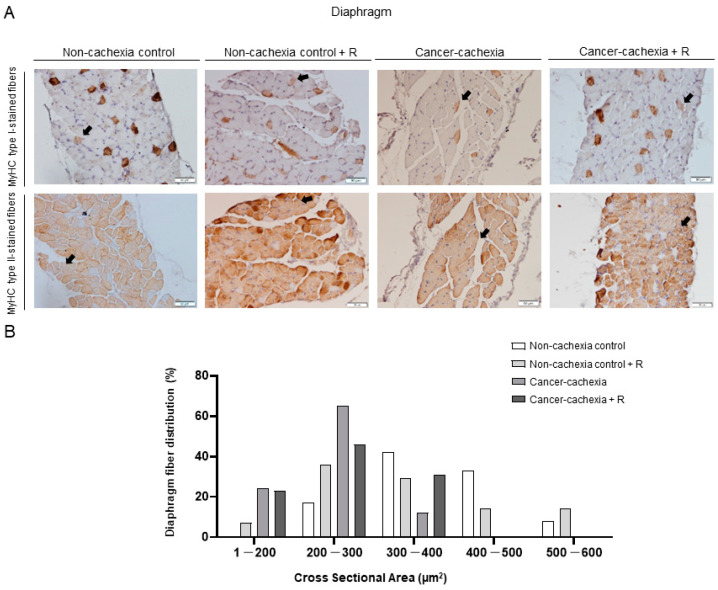
Representative images of diaphragm muscle fibers (**A**). Myofibers with anti-MyHC type I antibody staining (brown color) are shown in the top panel and anti-MyHC type II antibody staining in the middle panel. Hybrid fibers (arrows) are seen in both panels. Schematic representation of the distribution of the CSA of the muscle fibers in the diaphragm muscle (**B**). Abbreviations: R, rucaparib; MyHC, myosin heavy chain; CSA, cross-sectional area.

No significant differences were detected in the proportions of any fiber type in either the respiratory or limb muscles of the cancer cachexia mice with or without treatment with rucaparib (Figure 4A and Figure 5A and Table 2, respectively). In both the gastrocnemius and the diaphragm of cancer cachexia mice, the CSA of slow- and fast-twitch fibers and hybrid fibers was significantly lower than in non-cachectic controls (Figure 4A,B and Figure 5A,B and Table 2, respectively). Treatment of the cancer cachexia mice with rucaparib did not elicit any significant effect on the CSA of the cancer cachexia mice (Figure 4A,B and Figure 5A,B and Table 2, respectively).

### 3.5. Rucaparib Attenuated Proteolytic Markers in Cancer Cachexia Mice

**Figure 6:**
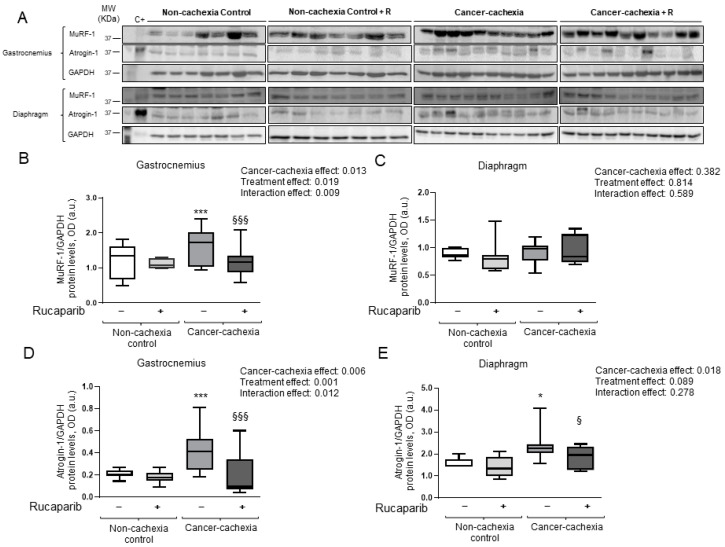
Immunoblots examples of MuRF-1, atrogin-1, and GAPDH proteins in the gastrocnemius and diaphragm muscles of all experimental groups of mice (**A**). Box plot representation of MuRF-1 protein levels in the gastrocnemius (**B**) and diaphragm (**C**) muscles of the different experimental groups of mice. Box plot representation of atrogin-1 protein levels in the gastrocnemius (**D**) and diaphragm (**E**) muscles of the different study groups of mice. Abbreviations: MuRF-1, muscle RING-finger protein-1; GAPDH, glyceraldehyde-3-phosphate dehydrogenase; MW, molecular weight; kDa, kilodalton; OD, optical densities; a.u., arbitrary units; −, untreated with rucaparib; +, treated with rucaparib. Statistical significance: * *p* ≤ 0.05 and *** *p* ≤ 0.001 between the untreated non-cachexia controls and the non-treated cancer cachexia mice; ^§^ *p* ≤ 0.05 and ^§§§^ *p* ≤ 0.001 between the cancer cachexia mice treated with rucaparib and the non-treated cancer cachexia mice. Two-way ANOVA significance (*p* value) of cancer cachexia and treatment and interaction effects are also shown for each variable. Uncropped WB images were shown in Figure S1.

**Figure 7:**
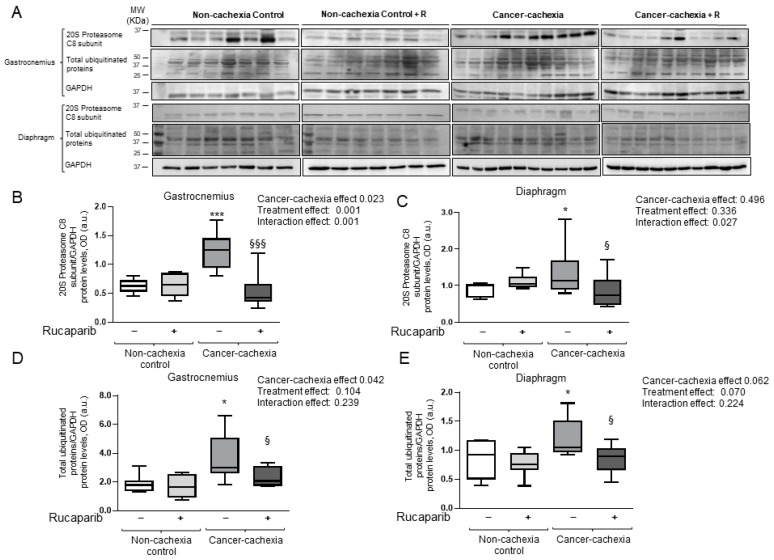
Immunoblot examples of 20S proteasome C8 subunit, total ubiquitinated proteins, and GAPDH proteins in the gastrocnemius and diaphragm muscles of all experimental groups of mice (**A**). Box plot representation of 20S proteasome C8 subunit protein levels in the gastrocnemius (**B**) and diaphragm (**C**) muscles of the different study groups of mice. Box plot representation of total ubiquitinated protein levels in the gastrocnemius (**D**) and diaphragm (**E**) muscles of the different study groups of mice. Abbreviations: GAPDH, glyceraldehyde-3-phosphate dehydrogenase; MW, molecular weight; kDa, kilodalton; OD, optical densities; a.u., arbitrary units; −, untreated with rucaparib; +, treated with rucaparib. Statistical significance: * *p* ≤ 0.05 and *** *p* ≤ 0.001 between the untreated non-cachexia controls and the non-treated cancer cachexia mice; ^§^ *p* ≤ 0.05 and ^§§§^ *p* ≤ 0.001 between the cancer cachexia mice treated with rucaparib and the non-treated cancer cachexia mice. Two-way ANOVA significance (*p* value) of cancer cachexia and treatment and interaction effects are also shown for each variable. Uncropped WB images were shown in Figure S1.

Muscle levels of the proteolytic marker MuRF-1 significantly increased in the gastrocnemius, but not in the diaphragm, of cancer cachexia mice compared to non-cachexia controls (Figure 6A–C, respectively). In respiratory and limb muscles, atrogin-1 levels were greater in the cancer cachexia mice than in the non-cachexia controls, and rucaparib significantly reduced those levels in the treated animals (Figure 6A,D,E, respectively). Furthermore, a significant rise in levels of 20S proteasome C8 subunit and total protein ubiquitination was detected in the diaphragm and gastrocnemius muscles of cancer cachexia mice compared to non-treated animals, and rucaparib elicited a significant decline in those levels in both muscle types (Figure 7A–E, respectively).

### 3.6. Effects of Rucaparib on Autophagy Markers in Respiratory and Limb Muscles

**Figure 8:**
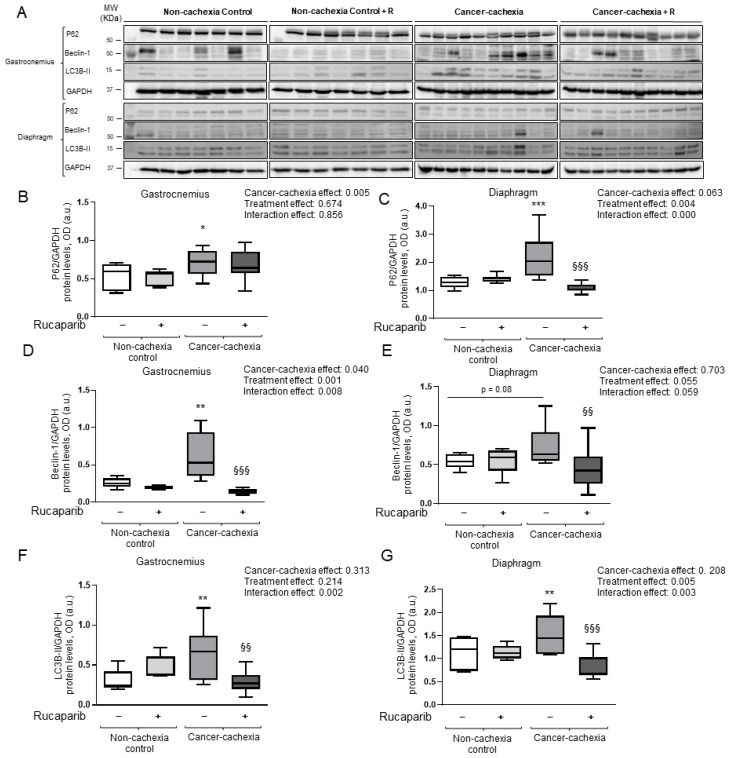
Immunoblots of p62, beclin-1, LC3B, and GAPDH proteins in the gastrocnemius and diaphragm muscles of all experimental groups of mice (**A**). Box plot representation of p62 protein levels in the gastrocnemius (**B**) and diaphragm (**C**) muscles of the different experimental groups of mice. Box plot representation of beclin-1 protein content in the gastrocnemius (**D**) and diaphragm (**E**) muscles of the different experimental groups of mice. Box plot representation of LC3B-II protein levels in the gastrocnemius (**F**) and diaphragm (**G**) muscles of the different study groups of mice. Abbreviations: LC3B, microtubule-associated protein 1 light chain 3B; p62, nucleoporin p62; GAPDH, glyceraldehyde-3-phosphate dehydrogenase; MW, molecular weight; kDa, kilodalton; OD, optical densities; a.u., arbitrary units; −, untreated with rucaparib; +, treated with rucaparib. Statistical significance: * *p* ≤ 0.05, ** *p* ≤ 0.01, and *** *p* ≤ 0.001 between the untreated non-cachexia controls and the non-treated cancer cachexia mice; ^§§^ *p* ≤ 0.01 and ^§§§^ *p* ≤ 0.001 between the cancer cachexia mice treated with rucaparib and the non-treated cancer cachexia mice. Two-way ANOVA significance (*p* value) of cancer cachexia and treatment and interaction effects are also shown for each variable. Uncropped WB images were shown in Figure S1.

A significant increase in the autophagy marker p62 was observed in both the gastrocnemius and diaphragm of cancer cachexia mice compared to non-cachectic controls, and rucaparib elicited a significant decline only in the diaphragm (Figure 8A–C, respectively). Protein levels of beclin-1 and LC3B were significantly greater in the gastrocnemius and diaphragm (*p* = 0.08, beclin-1) of cancer cachexia mice than in the non-cachectic controls, and treatment with rucaparib significantly reduced those levels in the cachectic animals (Figure 8A,D–G, respectively). Full Western blot figures can be found in the supplementary materials. 

## 4. Discussion

In the current study, a significant rise in muscle damage as identified using the reliable marker troponin I was observed in the systemic compartment of the cancer cachexia mice compared to the non-cachexia controls. Importantly, increased levels of troponin I significantly declined in the cachectic mice that received treatment with the PARP-1/2 inhibitor rucaparib. In addition, at the muscle level, structural abnormalities characterized by the presence of internal nuclei and inflammatory and necrotic cells were significantly greater in both the diaphragm and gastrocnemius muscles of the cancer cachexia mice compared to the controls, and the rise in inflammatory cells was of a greater magnitude in the limb muscle. Interestingly, concomitant treatment with rucaparib elicited a significant decline in the analyzed parameters except for the inflammatory cell counts in the respiratory muscle of the cachectic mice, where levels for all groups were lower than those observed in the limb muscle. The presence of inflammatory cells may vary across muscle types within the same experimental model and animal type as also previously shown in models of hypoxia [56] and ischemia–reperfusion in rodents [57]. These findings reveal that PARP activity underlies muscle’s structural damage in this model of cancer cachexia. Rucaparib elicited significant effects only in muscles of the cachectic mice but not in those of the control rodents.

Previous results demonstrated that the absence of PARP-1 activity led to an improvement in muscle structural abnormalities as well as in other events such as apoptosis, atrophy, and inflammation in a model of rotator cuff tear in mice [40]. Moreover, muscle regeneration following injury was significantly faster in the mice with PARP-1 genetic deletion [40]. Of note, pharmacological PARP1/2 inhibition in LLC-PK1 cells (porcine kidney cells) elicited apoptosis within the cells with higher levels of oxidants following exposure to hydrogen peroxide [58]. In another investigation based on a mouse model of ischemia/reperfusion [57], tissue viability was preserved along with a reduction in inflammatory cell counts in the hindlimb muscles following treatment with a PARP-1/2 pharmacological inhibitor. Collectively, these results indicate that PARP activity inhibition mitigates muscle damage, while improving muscle structure [40,57,58]. Thus, the results obtained in the present study are aligned with previously published observations [40,57,58].

A rise in the expression of markers of the ubiquitin–proteasome system was observed in the cachectic muscles compared to the non-cachectic controls. Importantly, treatment with rucaparib elicited a significant decline in levels of atrogin-1, 20S proteasome C8 subunit, and total protein ubiquitination in both the diaphragm and gastrocnemius muscles of the treated cachectic mice. Moreover, MuRF-1 protein levels also significantly decreased in the limb muscle of the treated mice. These findings are in agreement with previous results in which genetic deletion of either PARP-1 or PARP-2 elicited a decline in the levels of muscle proteolytic markers in lung-cancer-induced cachexia [17].

Although the loss in muscle weights and the reduction in CSA of both slow- and fast-twitch fibers in respiratory and limb muscles was attenuated as a result of treatment with rucaparib, a statistically significant improvement was not detected in any of those parameters in the cachectic mice. Furthermore, treatment with rucaparib did not rescue muscle force in the treated animals. Taken together, these findings suggest that rucaparib was not able to significantly restore muscle weight or function in this model of cancer-induced cachexia. Physical activity, however, significantly improved in the cachectic mice treated with rucaparib. Previous results also demonstrated that inhibition of PARP-1 may result in a better skeletal muscle performance in models of disuse-induced atrophy [59]. Another seminal study also revealed that skeletal muscle dysfunction was attenuated by PARP inhibition, probably as a result of mitochondrial oxidative capacity boosting [60]. In the current investigation, troponin I levels inversely correlated with grip strength measurements in both groups of cachectic mice. Although the correlation was weaker in the treated mice, the inhibition of PARP activity elicited by rucaparib did not blunt such a relationship. Collectively, these findings along with those encountered in the present study reveal that PARP activity inhibition rather improves physical activity and performance with reference to muscle mass or strength.

Autophagy, which is a lysosome-dependent degradative pathway, is highly activated in cancer cells as a result of chemotherapy and/or radiation. In the current study, expression of the autophagy markers LC3B and beclin-1 increased in both the respiratory and limb muscles of the cancer-cachectic mice, and treatment with rucaparib elicited a significant decline in the same muscles. Additionally, protein levels of p62 rose in the diaphragm and gastrocnemius of the cancer-cachectic mice, but a significant decrease in those levels was only seen in the respiratory muscle following treatment with rucaparib. These findings are in line with previous results, in which PARP-2 activity was shown to induce autophagy in cultured myoblasts [61]. Hence, inhibition of PARP lessens autophagy in skeletal muscles.

### Study Limitations

Tumor burden significantly declined in the cachectic mice treated with rucaparib. Although beneficial effects might be expected from such a decrease (28%), no significant effects on either muscle weight or function were observed in the studied muscles. These findings suggest that the muscle damage attenuation and improvement was most likely related to the effects of PARP activity inhibition on both respiratory and limb muscles of the treated cachectic mice.

Another limitation might be related to the animal experimental model and to what extent these results can be applied to clinical settings. Nonetheless, we believe that the results obtained in the present study can serve as the basis to better understand the mechanisms whereby PARP may be involved in the pathophysiology of cancer cachexia.

## 5. Conclusions

Pharmacological inhibition of PARP activity did not exert any significant improvements in muscle weight, fiber size, or limb muscle strength. Treatment with rucaparib, however, elicited a significant improvement in muscle damage and structural abnormalities and physical activity in cancer-cachectic mice. These findings suggest that rucaparib exerts its beneficial effects on cancer cachexia performance through the restoration of muscle structure. The results obtained in the present study can serve as the basis to better understand the mechanisms whereby PARP may be involved in the pathophysiology of cancer cachexia.

## Figures and Tables

**Table 1:** Physiological parameters in all experimental groups of mice.

	Non-Cachexia Controls		Cancer Cachexia		Cancer Cachexia Effect 7 (*p* Value)	Treatment Effect (*p* Value)	Interaction Effect (*p* Value)
Rucaparib	−	+	−	+			
Initial body weight (g)	21.26 (0.77)	20.76 (0.88)	20.89 (0.77)	20.45 (0.77)	0.876	0.985	0.194
Final body weight (g)	21.43 (0.64)	20.31 (1.03)	19.77 (1.61) *	20.91 (2.01)	0.316	0.979	0.037
Final body weight without tumor (g)	21.43 (0.64)	20.31 (1.03)	18.36 (1.75) **	19.57 (2.38)	0.003	0.941	0.057
Body weight gain (%)	+0.79 (2.98)	−1.80 (2.04)	−7.40 (5.90) **	+2.52 (8.71) ^§§^	0.009	0.273	0.020
Body weight gain without tumor (%)	+0.79 (2.98)	−1.80 (2.04)	−12.11 (7.91) ***	−4.08 (10.69) ^§§^	0.007	0.335	0.042
Tumor weight (g)	NA	NA	1.70 (0.38)	1.21 (0.53) ^§^	NA	NA	NA
Tumor area (mm^2^)	NA	NA	1860.93 (506.80)	1296.75 (490.27) ^§^	NA	NA	NA
Diaphragm weight (g)	0.08 (0.01)	0.07 (0.03)	0.05 (0.01) ***	0.06 (0.01)	0.001	0.919	0.030
Gastrocnemius weight (g)	0.12 (0.01)	0.11 (0.02)	0.10 (0.02) **	0.11 (0.02)	0.004	0.741	0.202
Four-limb strength (g)/bw (g)	12.01 (0.89)	12.56 (1.06)	9.59 (1.50) ***	8.60 (1.85)	0.000	0.657	0.129
Four-limb grip strength gain (%)	+10.84 (5.17)	+14.04 (15.81)	−16.59 (11.09) ***	−17.41 (13.88)	0.000	0.567	0.214
Locomotor movements	236.58 (67.55)	223.98 (58.92)	117.2 (30.2) ***	200.81 (71.59) ^§§§^	0.000	0.000	0.000

Data are presented as means (SD). Abbreviations: −, untreated with rucaparib, +, treated with rucaparib; NA, not applicable; bw, body weight. Statistical significance: * *p* ≤ 0.05, ** *p* ≤ 0.01, and *** *p* ≤ 0.001 between the untreated non-cachexia controls and the non-treated cancer cachexia mice; ^§^ *p* ≤ 0.05, ^§§^ *p* ≤ 0.01, and ^§§§^ *p* ≤ 0.001 between the cancer cachexia mice treated with rucaparib and the non-treated cancer cachexia mice. Two-way ANOVA significance (*p* value) of cancer cachexia and treatment and interaction effects are also shown for each variable. Statistically significant differences are shaded in the table.

**Table 2:** Muscle fiber morphometric features of the gastrocnemius and diaphragm muscles in the experimental groups.

		Non-Cachexia Controls		Cancer Cachexia		Cancer Cachexia Effect (*p* Value)	Treatment Effect (*p* Value)	Interaction Effect (*p* Value)
	Rucaparib	−	+	−	+			
**Fiber Type Composition**								
**Proportion of fiber type, %**								
Type I fibers	Gastrocnemius	11.4 (3.6)	14.0 (3.3)	10.1 (2.1)	11.2 (2.5)	0.107	0.181	0.602
	Diaphragm	6.3 (1.7)	7.1 (1.2)	7.1 (2.9)	9.3 (2.1)	0.156	0.110	0.474
Type II fibers	Gastrocnemius	87.3 (3.4)	85.3 (3.2)	89.1 (1.9)	88.2 (2.4)	0.050	0.177	0.600
	Diaphragm	92.4 (1.7)	92.2 (0.9)	91.4 (3.4)	90.1 (1.8)	0.187	0.701	0.736
Hybrid fibers	Gastrocnemius	1.3 (0.9)	0.8 (0.8)	0.8 (0.9)	0.6 (0.6)	0.290	0.318	0.632
	Diaphragm	1.3 (0.5)	0.9 (0.2)	1.1 (0.3)	0.7 (0.3)	0.302	0.232	0.723
**Fiber cross-sectional area, µm^2^**								
Type I fibers	Gastrocnemius	758.1 (220.7)	762.3 (132.5)	460 (109.1) **	539.3 (123.3)	0.001	0.518	0.561
	Diaphragm	336.5 (72.8)	300.5 (85.0)	219.4 (40.2) **	226.2 (42.4)	0.009	0.074	0.010
Type II fibers	Gastrocnemius	848.0 (146.5)	884.4 (321.0)	592.9 (95.4) *	603.0 (135.7)	0.001	0.749	0.855
	Diaphragm	418.9 (63.7)	397.4 (76.5)	271.8 (48.3) ***	307.9 (24.6)	0.000	0.772	0.274
Hybrid fibers	Gastrocnemius	568.6 (174)	540.7 (138.6)	314.5 (132.2) *	432.1 (83.4)	0.024	0.536	0.324
	Diaphragm	351.2 (78.8)	335.8 (167.9)	197.5 (16.9) *	177.8 (27.2)	0.007	0.722	0.964

Data are presented as means (SD). Abbreviations: −, untreated with rucaparib, +, treated with rucaparib. Statistical significance: * *p* ≤ 0.05, ** *p* ≤ 0.01, and *** *p* ≤ 0.001 between the untreated non-cachexia controls and the non-treated cancer cachexia mice. Two-way ANOVA significance (*p* value) of cancer cachexia and treatment and interaction effects are also shown for each variable. Statistically significant differences are shaded in the table.

## Data Availability

The datasets generated and analyzed during the current study are available from the corresponding author on reasonable request.

## Supplementary Materials

The following supporting information can be downloaded at: https://www.mdpi.com/article/10.3390/cancers14122894/s1, Figure S1: uncropped WB images.
