# Supplementary material for: Attenuation of Muscle Damage, Structural Abnormalities, and Physical Activity in Respiratory and Limb Muscles following Treatment with Rucaparib in Lung Cancer Cachexia Mice
Source: Cancers (Basel). 2022 Jun 11;14(12):2894. doi: 10.3390/cancers14122894 (PMC9221243; doi:10.3390/cancers14122894)

Figure S1: uncropped WB images.

MURF-1 (40 kDa), corresponding to Figure 6A

Gastrocnemius

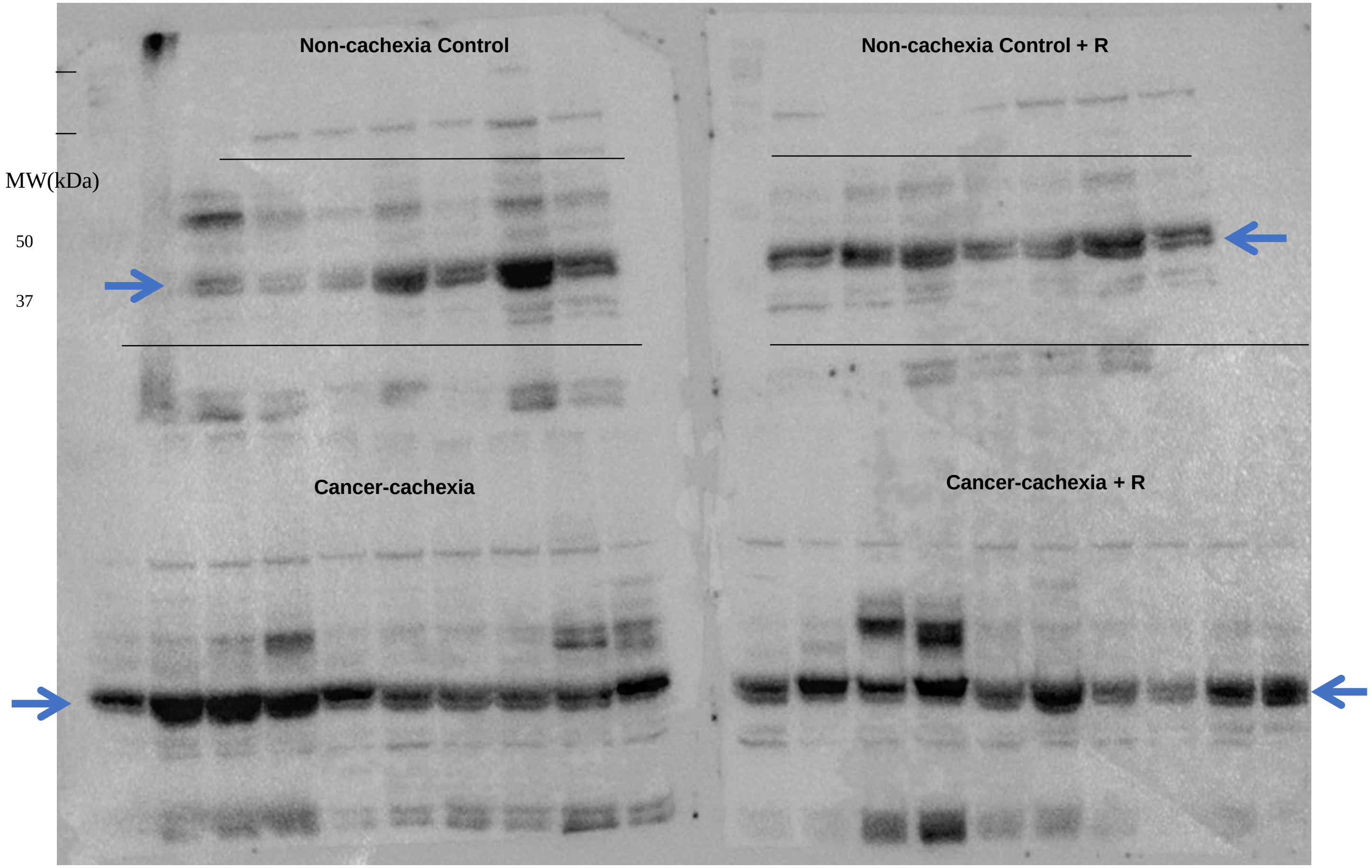

Atrogin-1 (42 kDa), corresponding to Figure 6A

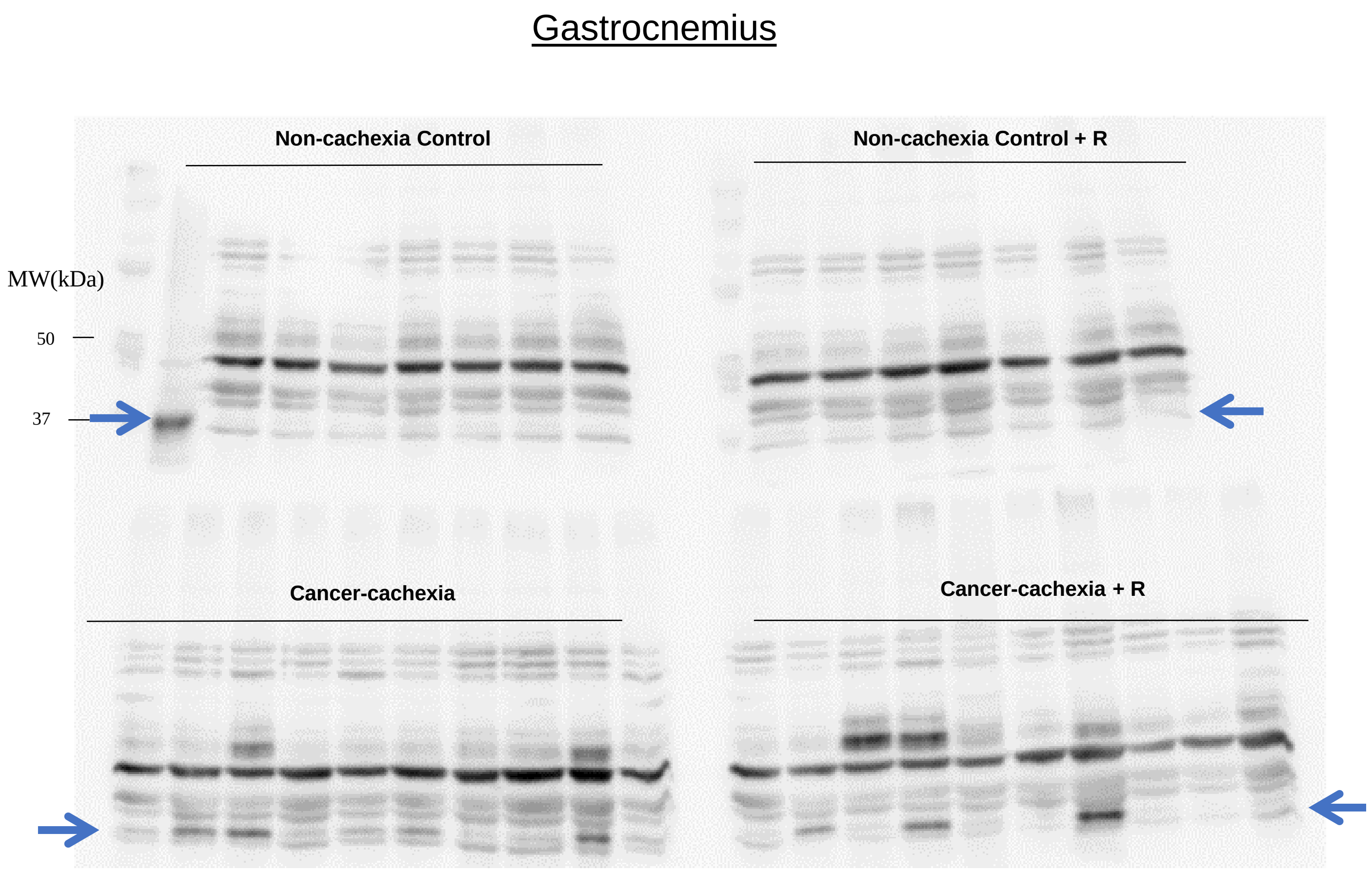

GAPDH (37 kDa), corresponding to Figure 6A

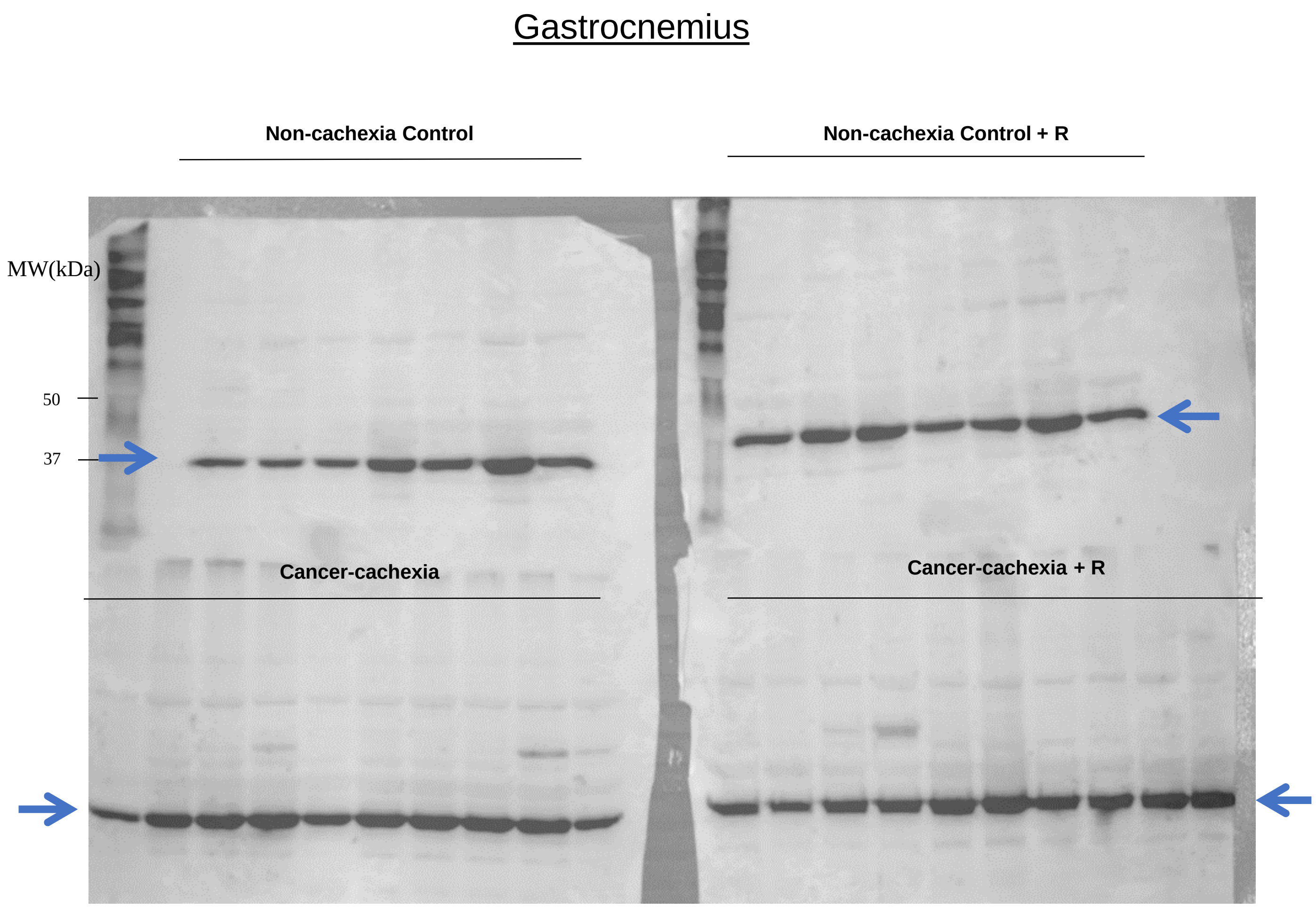

MURF-1 (40 kDa), corresponding to Figure 6A

Diaphragm

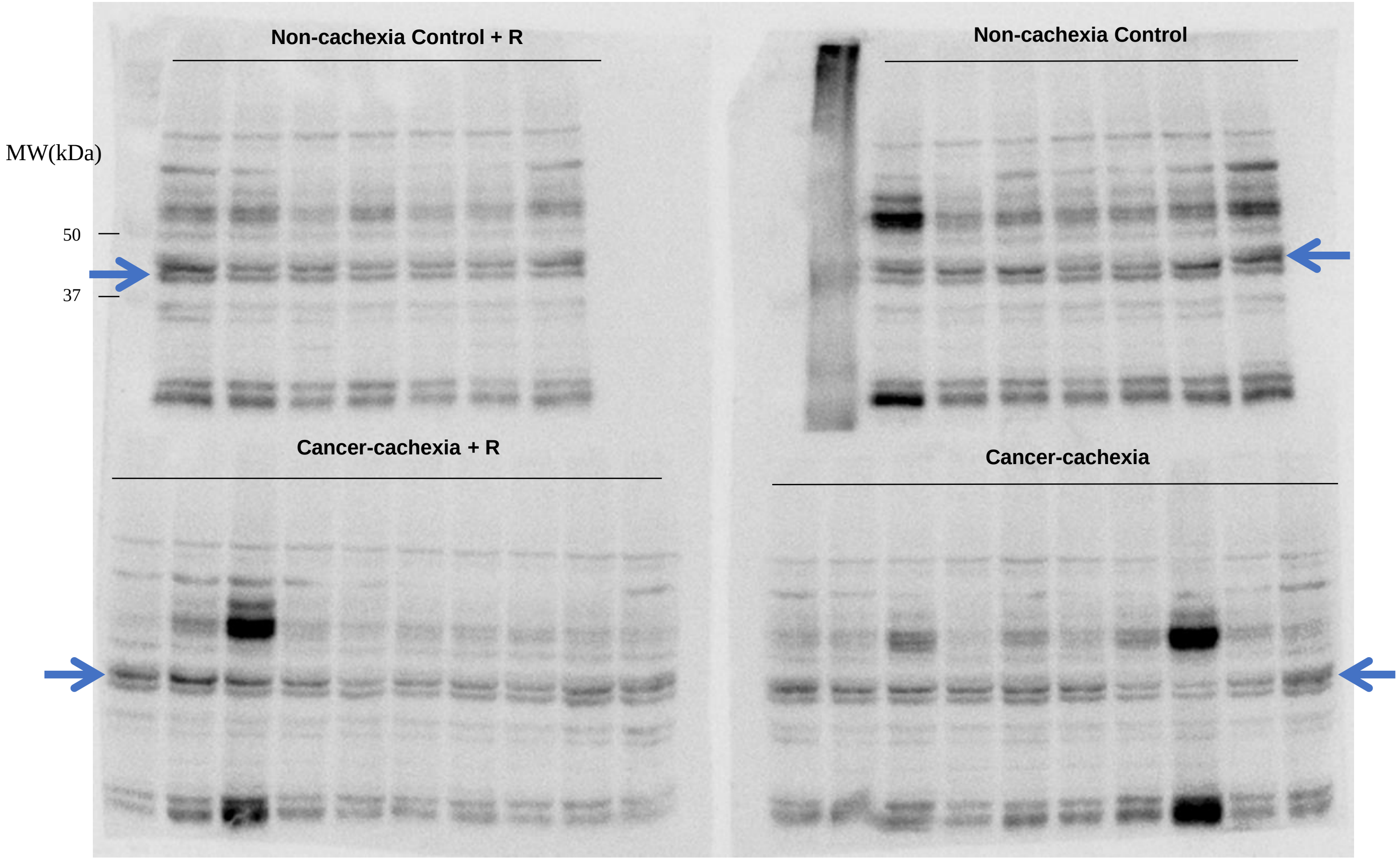

Atrogin-1 (42 kDa), corresponding to Figure 6A

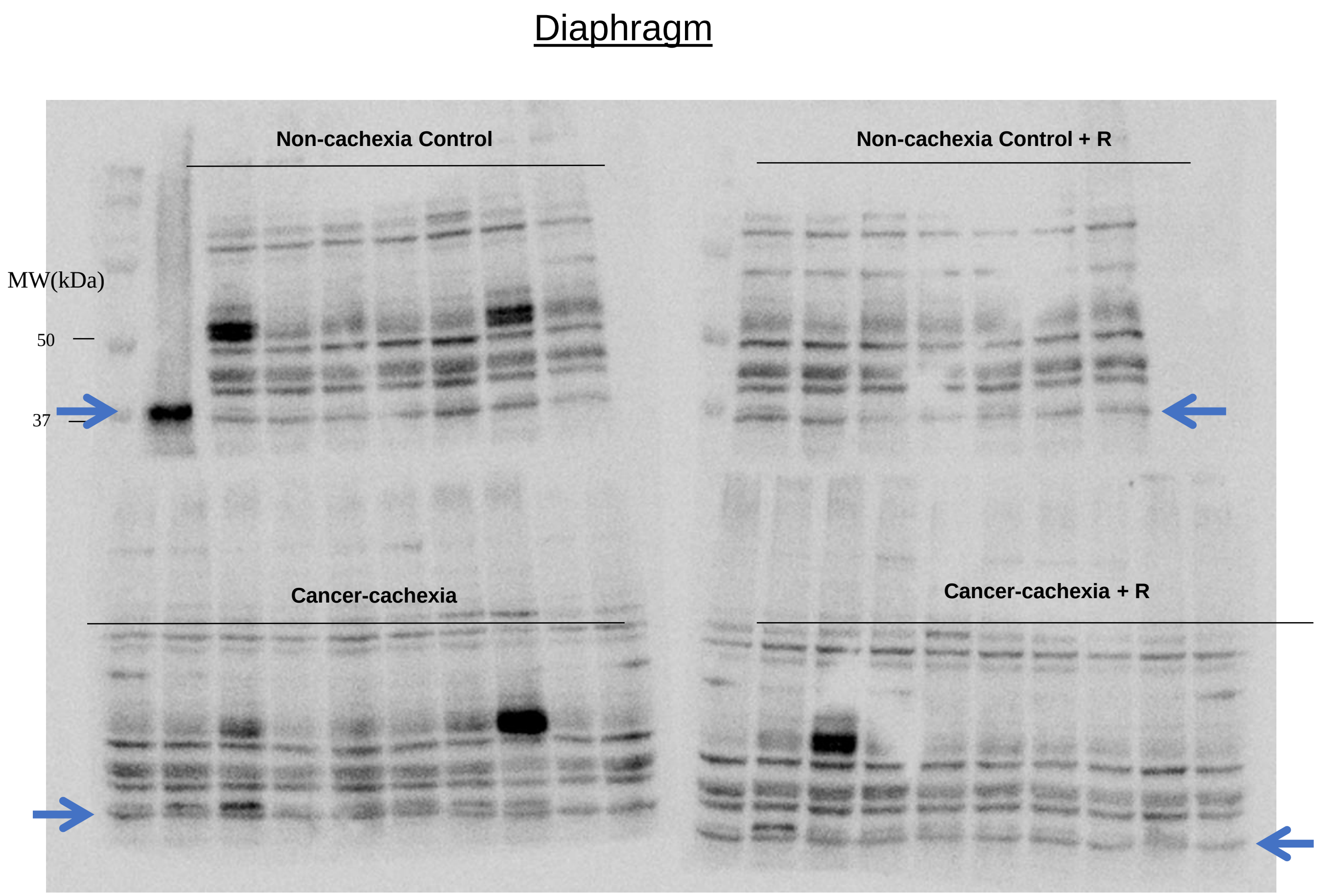

GAPDH (37 kDa), corresponding to Figure 6A

Diaphragm

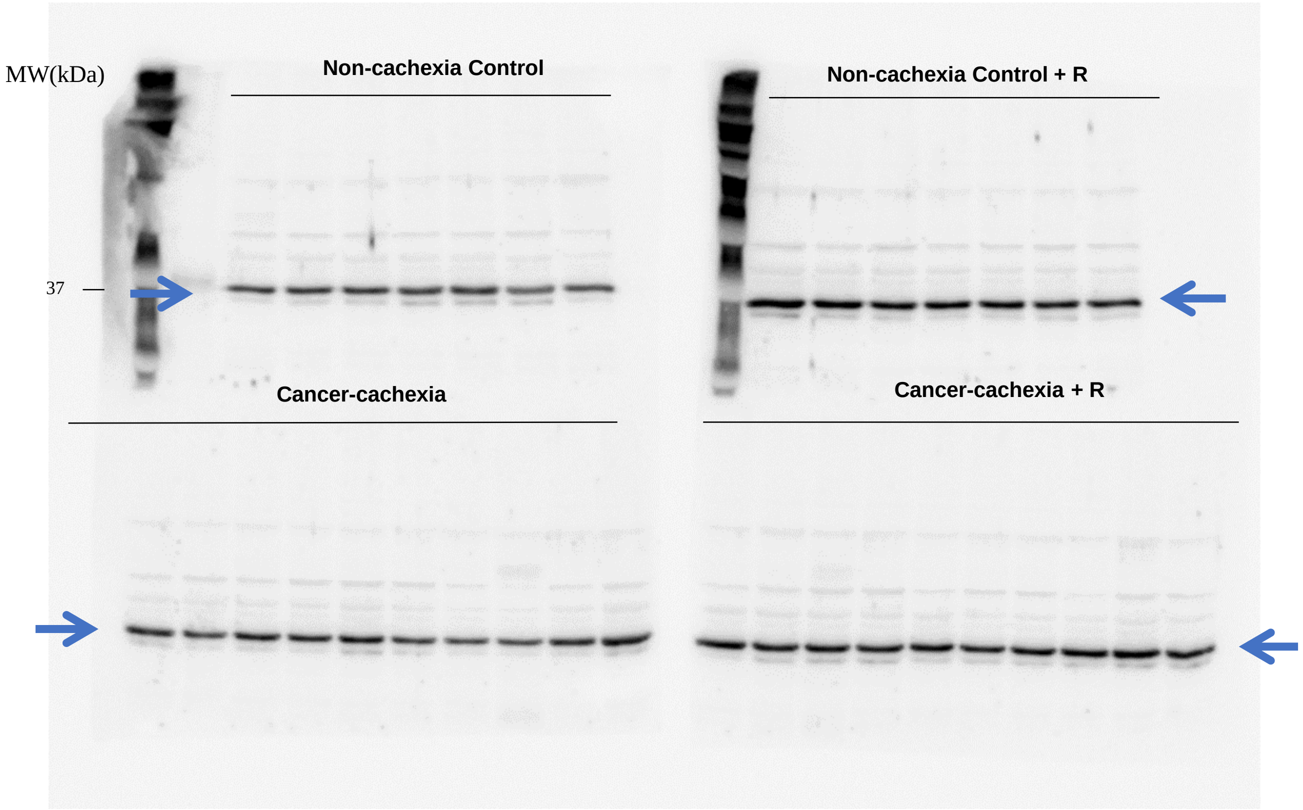

20S Proteosome C8 (30 kDa), corresponding to Figure 7A

Gastrocnemius

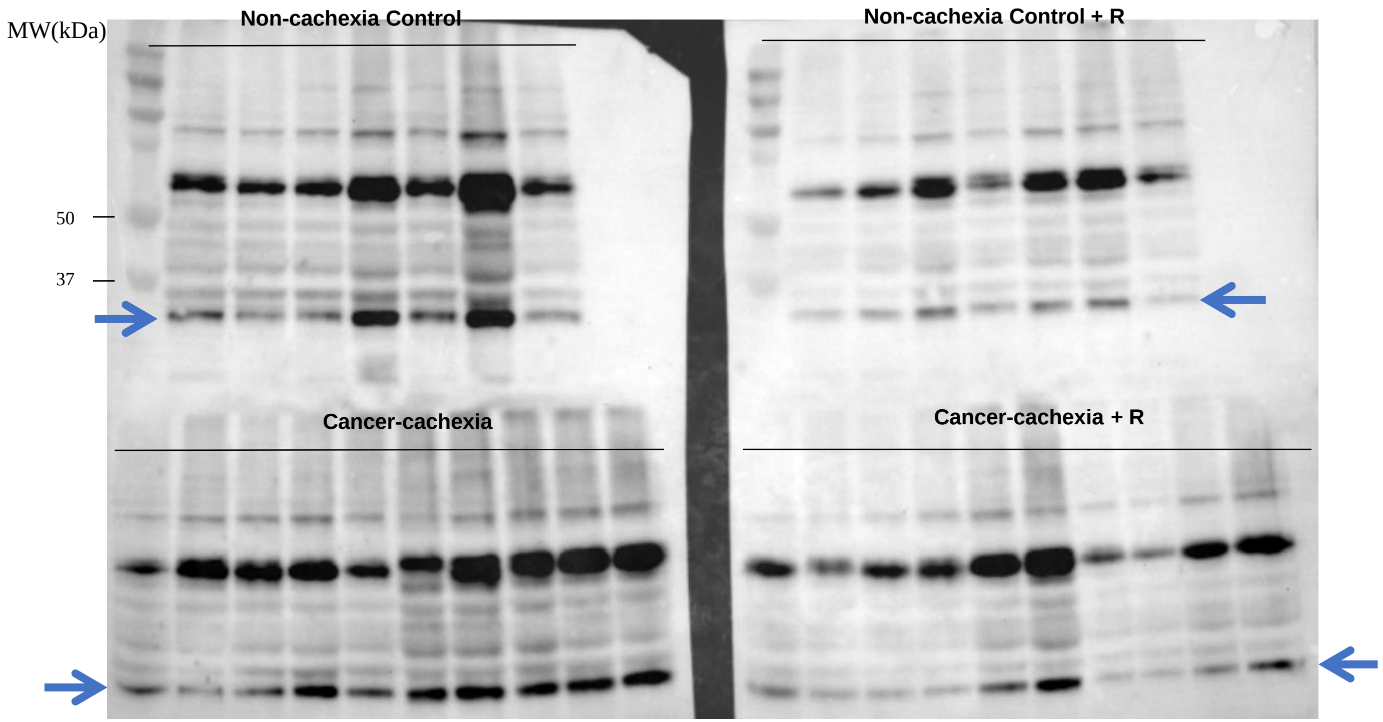

Total ubiquitinated proteins (multiple bands), corresponding to Figure 7A

Gastrocnemius

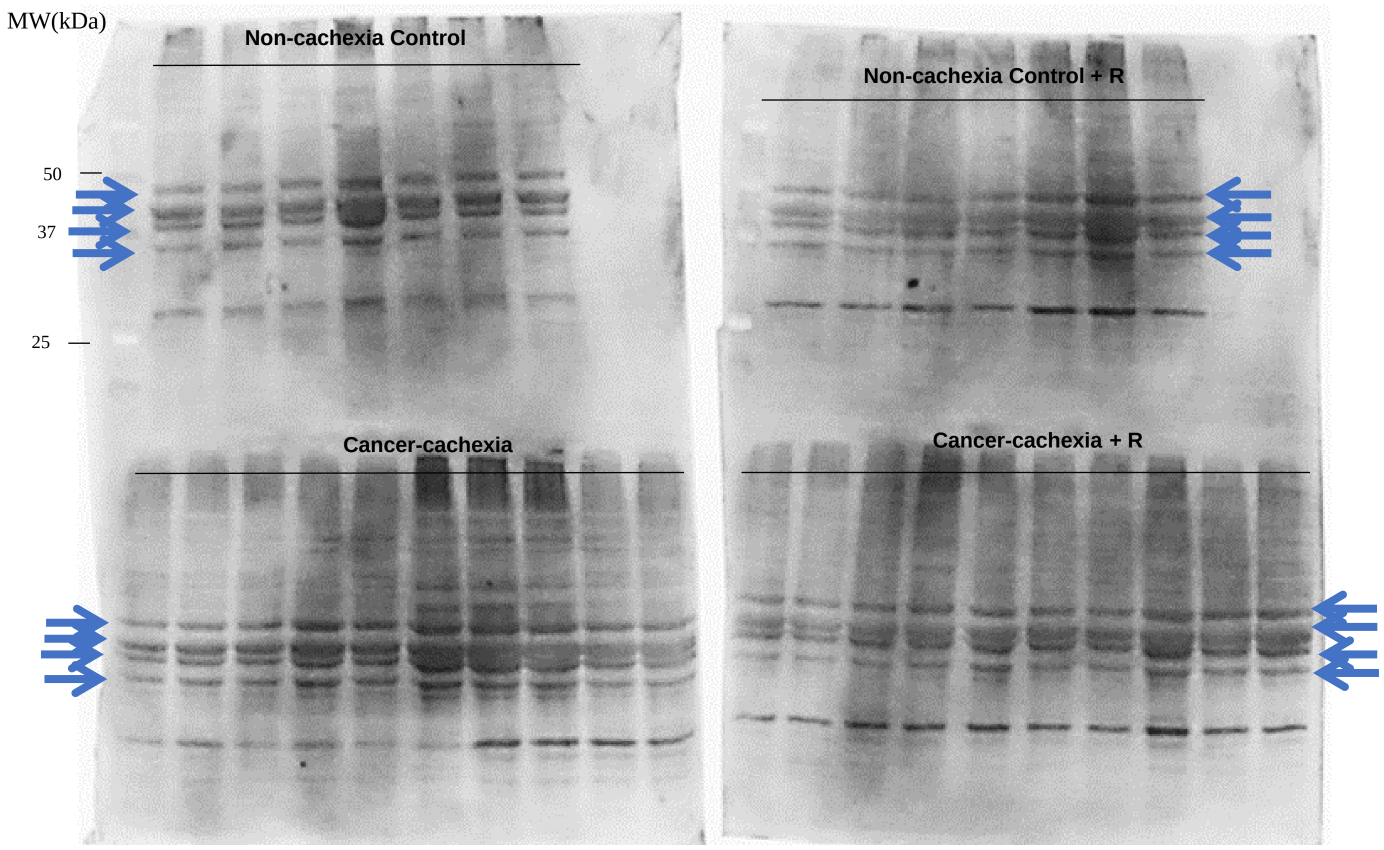

GAPDH (37 kDa), corresponding to Figure 7A

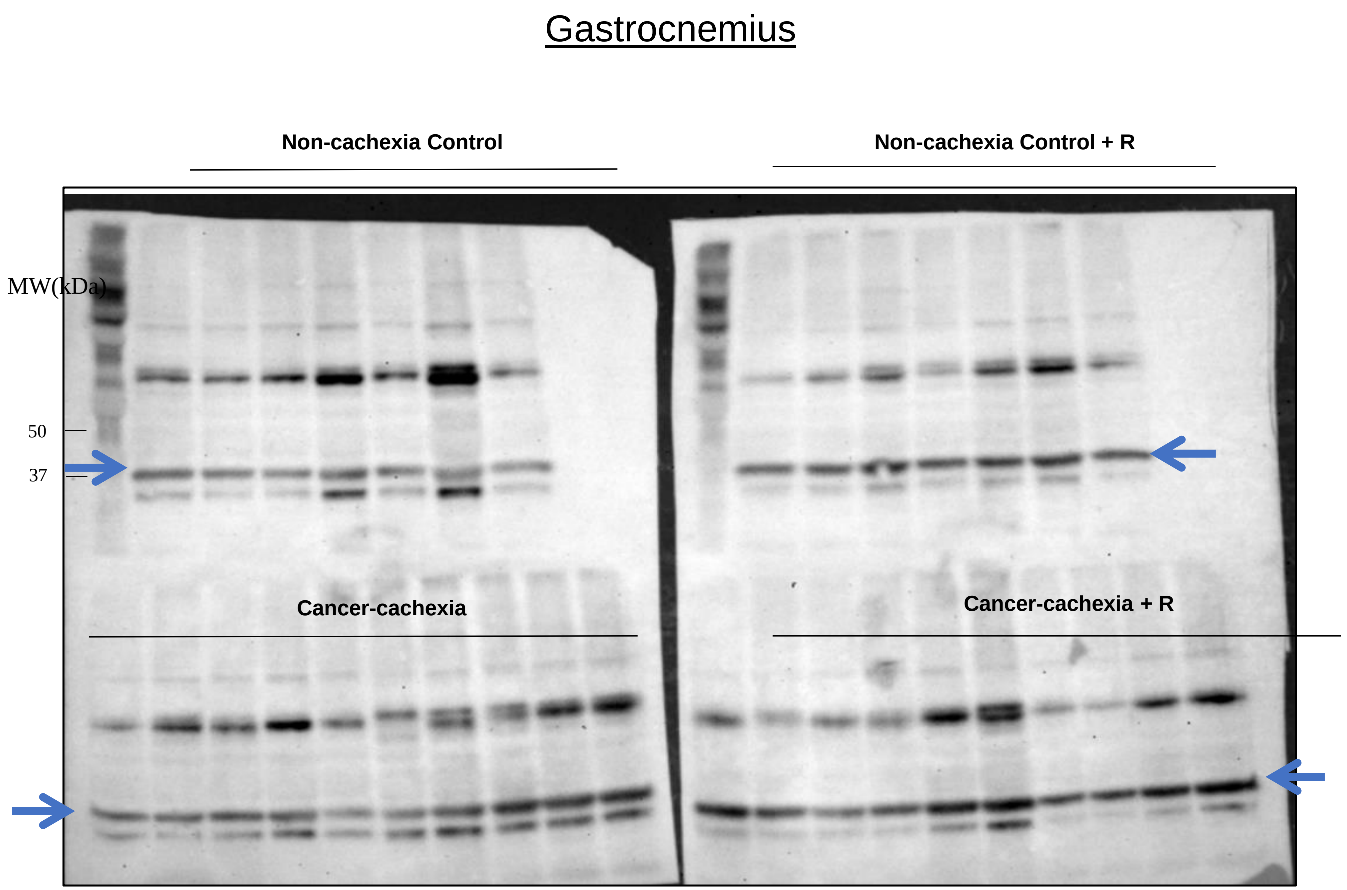

20S Proteosome C8 (30 kDa), corresponding to Figure 7A

Diaphragm

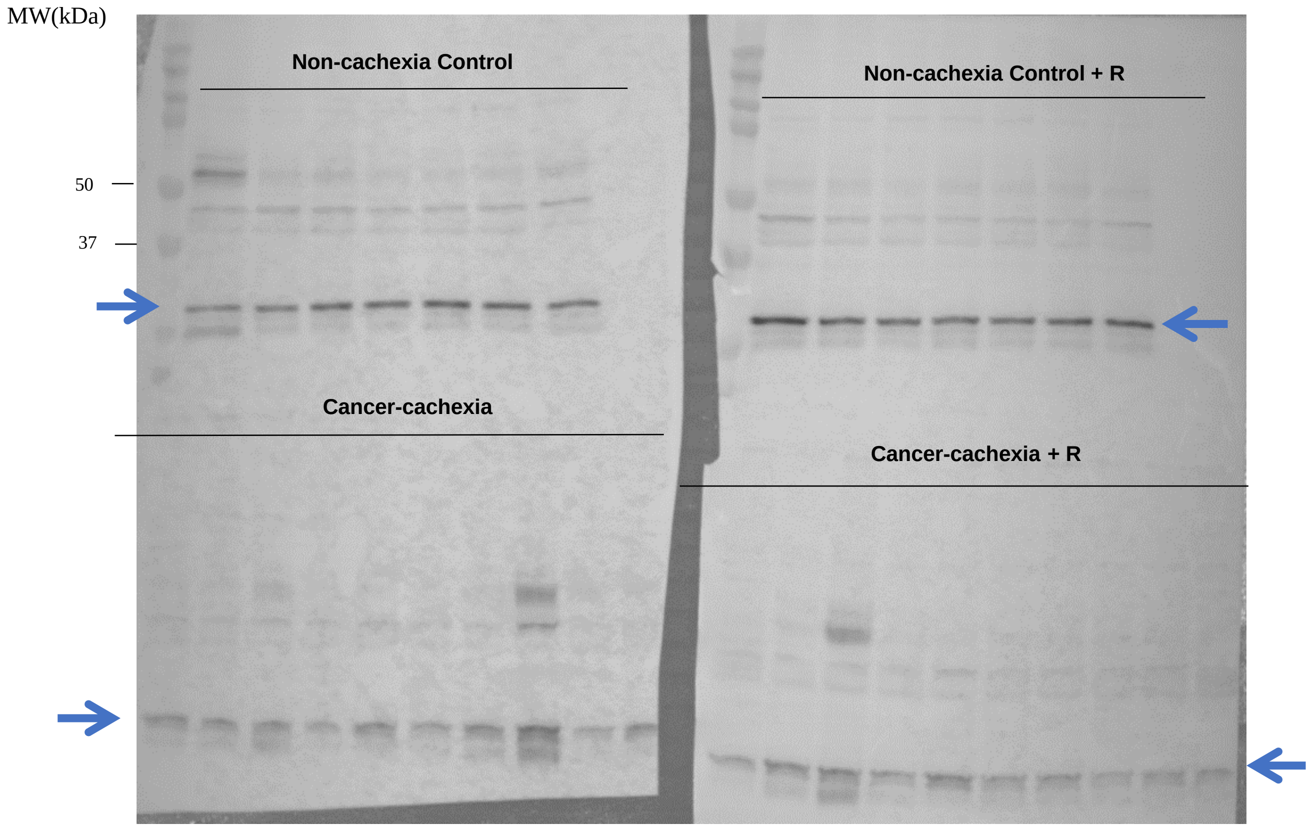

Total ubiquitinated proteins (multiple bands), corresponding to Figure 7A

Diaphragm

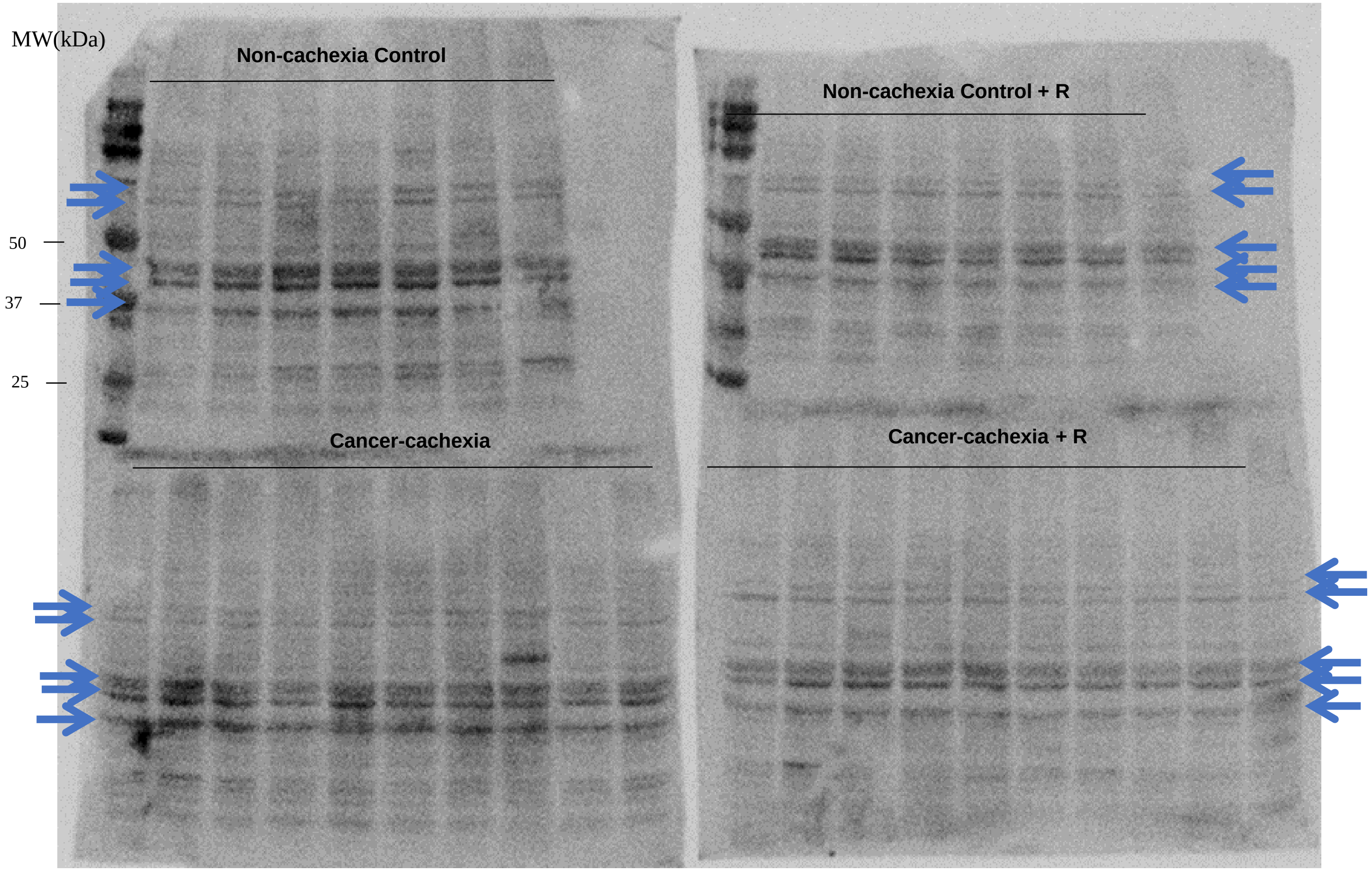

GAPDH (37 kDa), corresponding to Figure 7A

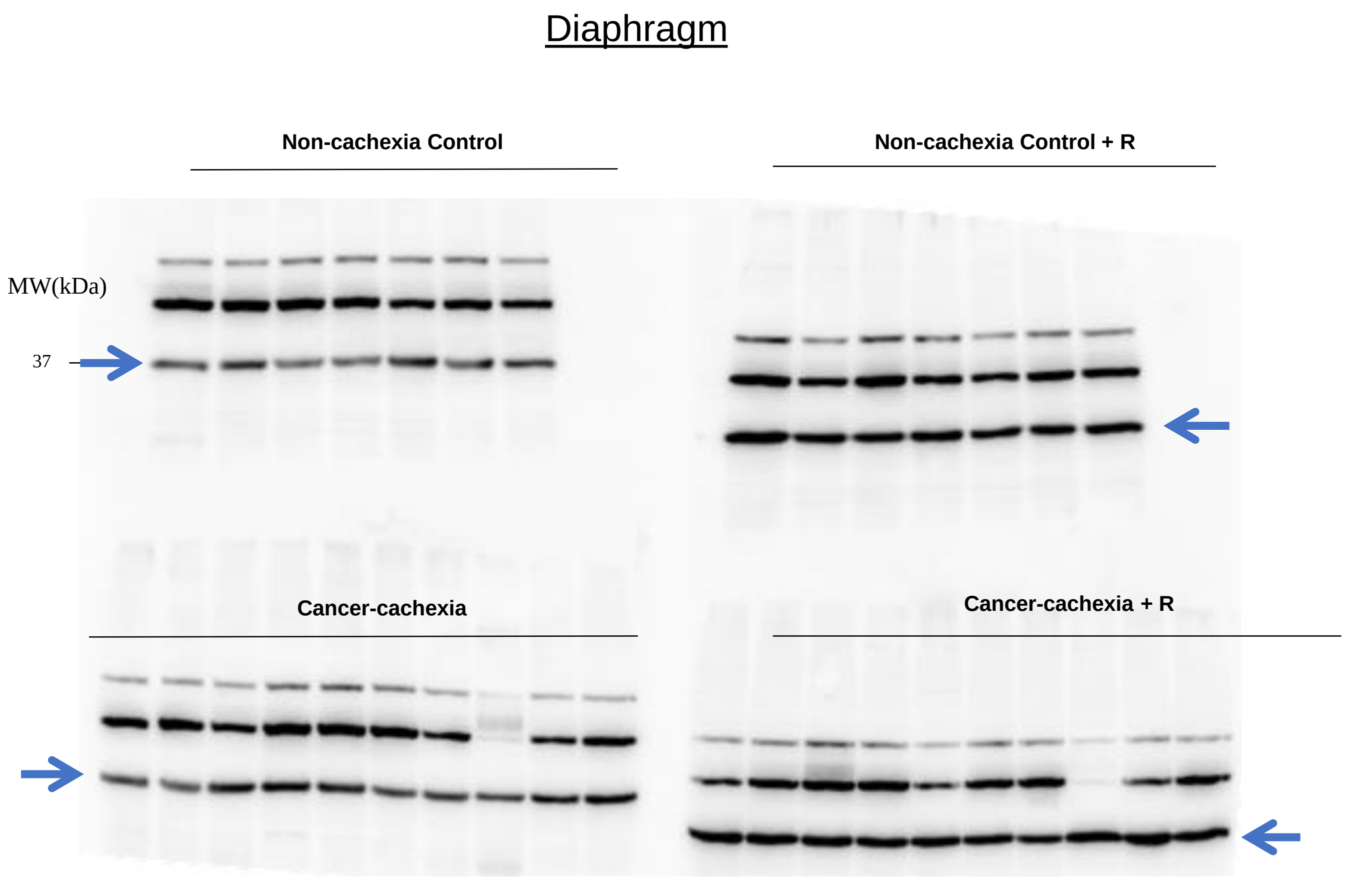

P62 (62 kDa), corresponding to Figure 8A

Gastrocnemius

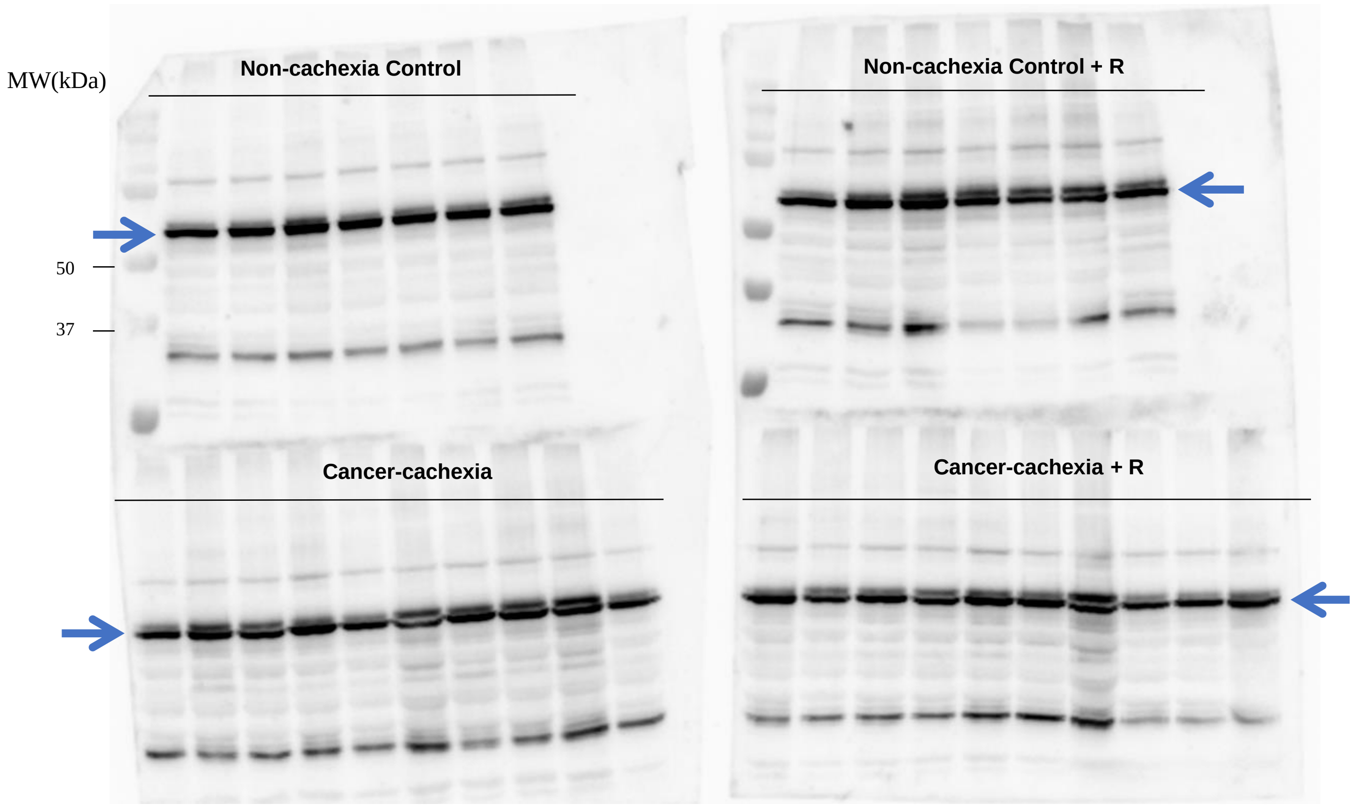

Beclin-1 (60 kDa), corresponding to Figure 8A

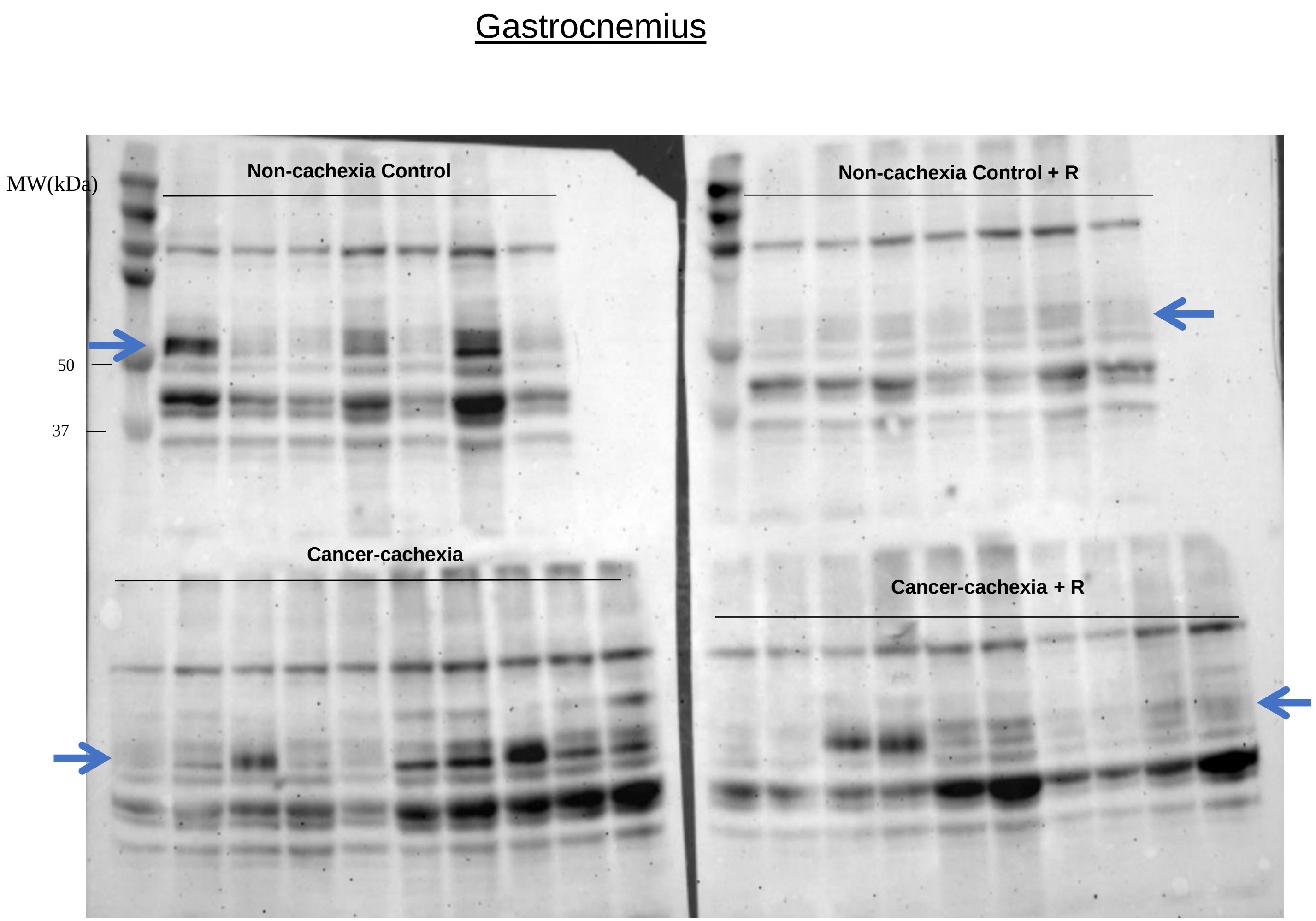

LC3B-II (14 kDa), corresponding to Figure 8A

Gastrocnemius

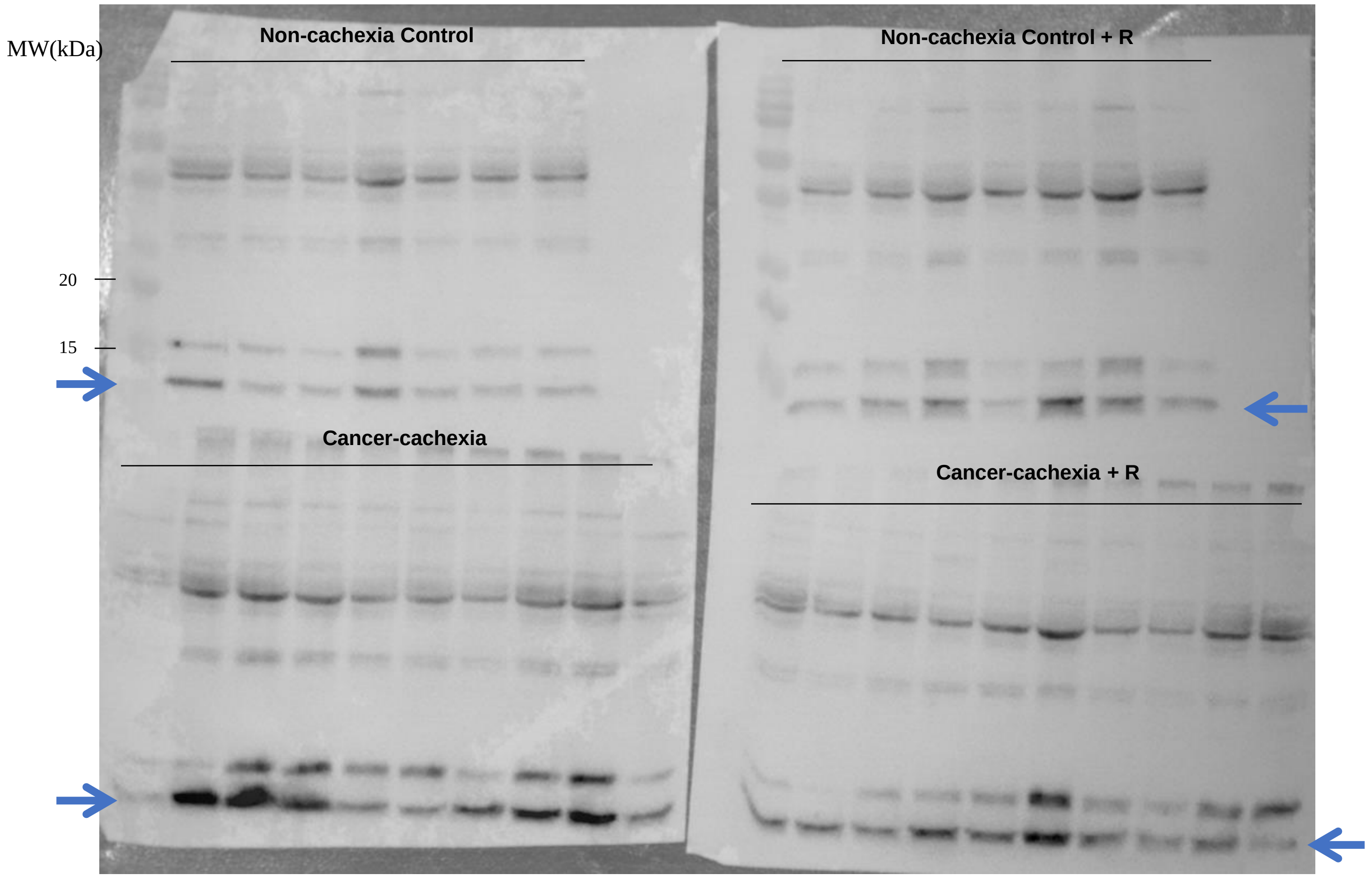

GAPDH (37 kDa), corresponding to Figure 8A

Gastrocnemius

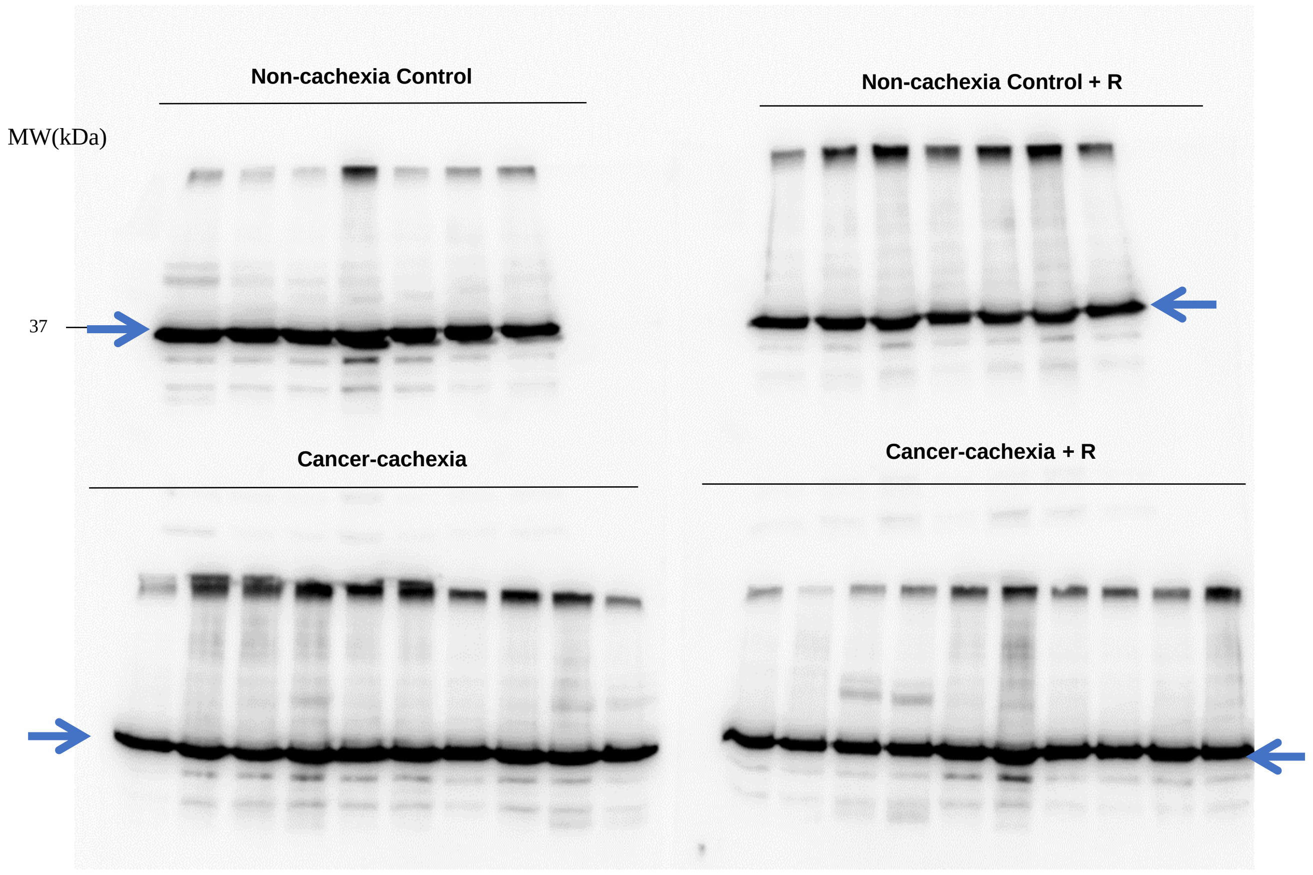

P62 (62 kDa), corresponding to Figure 8A

Diaphragm

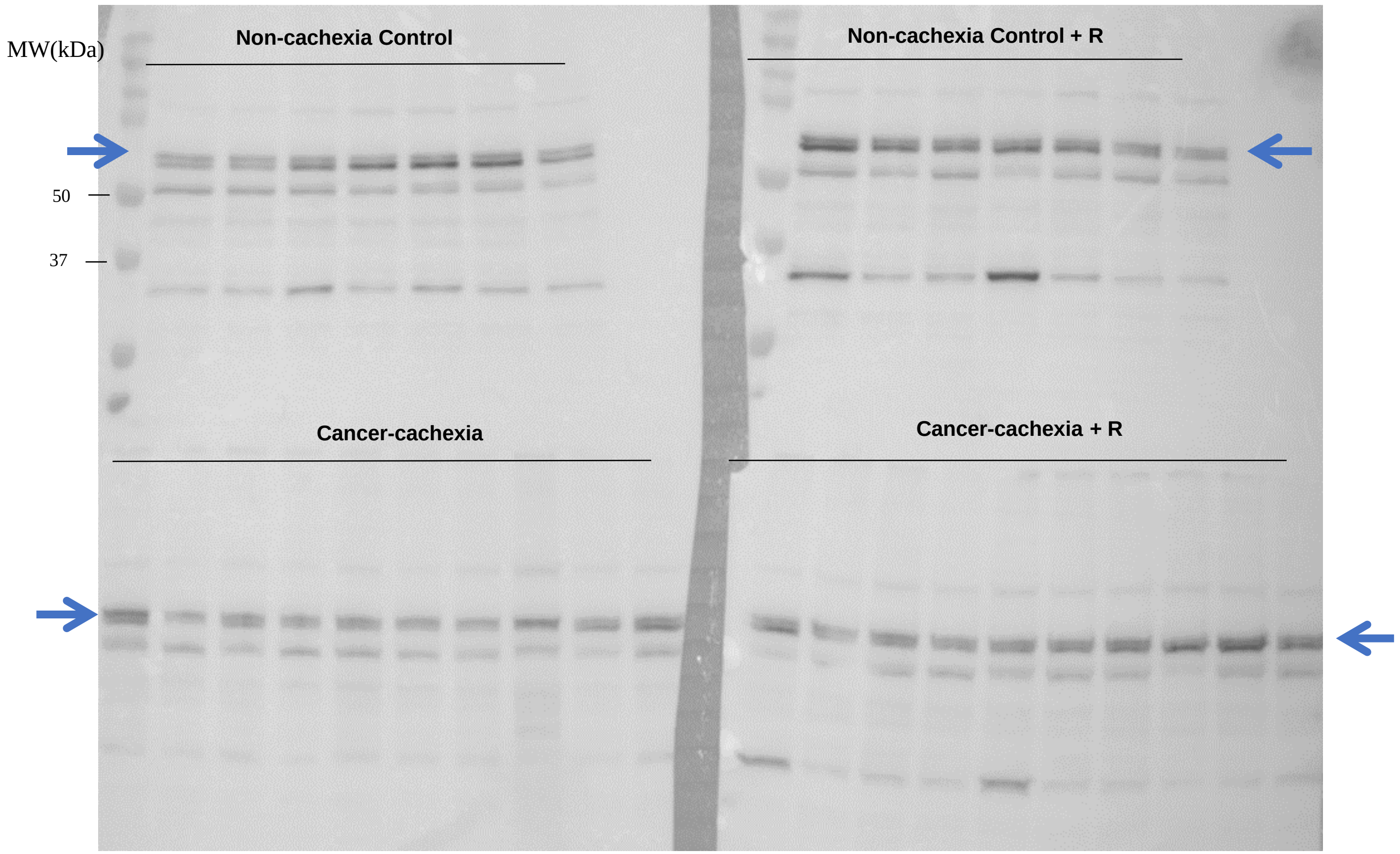

Beclin-1 (60 kDa), corresponding to Figure 8A

Diaphragm

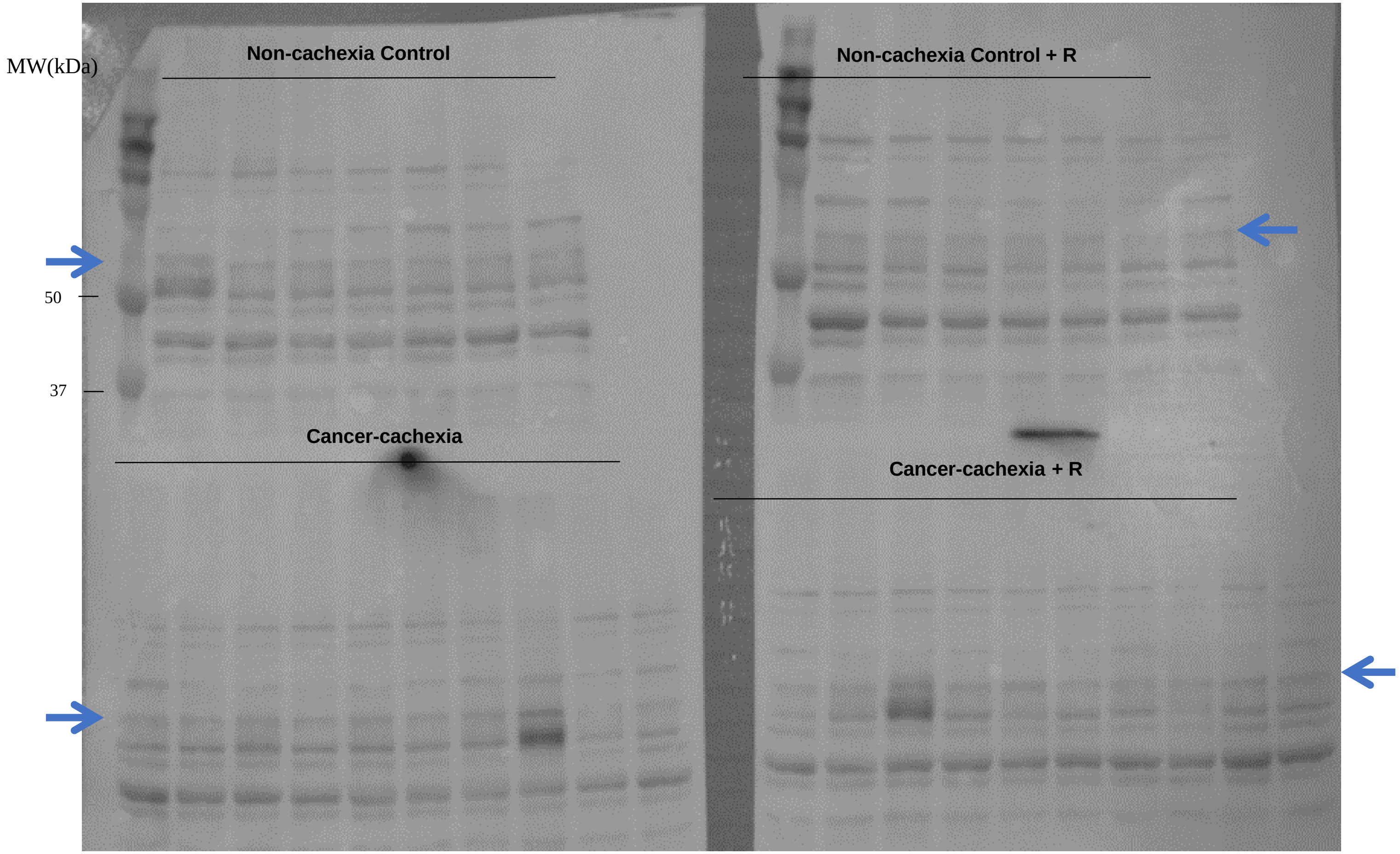

LC3B-II (14 kDa), corresponding to Figure 8A

Diaphragm

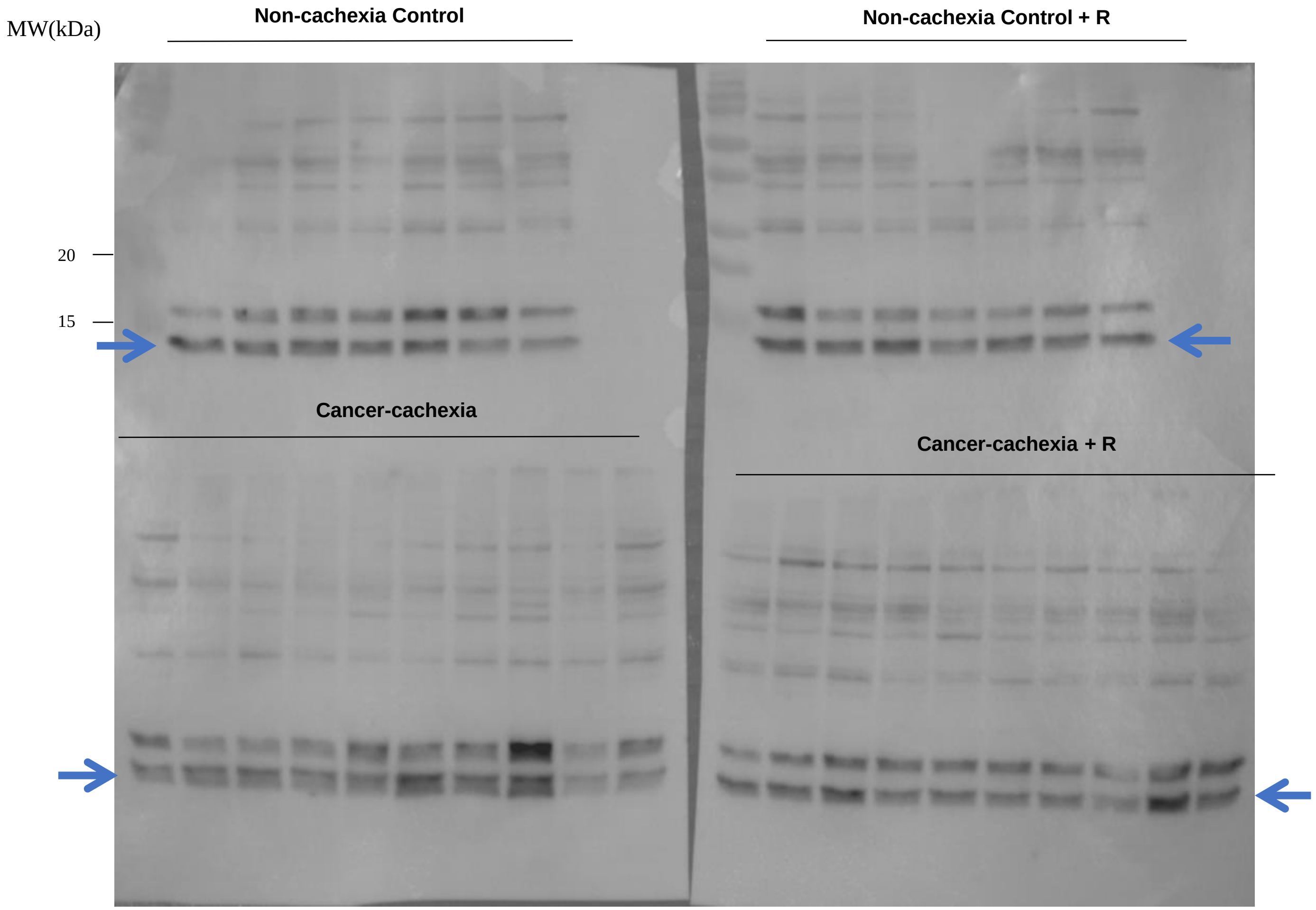

GAPDH (37 kDa), corresponding to Figure 8A

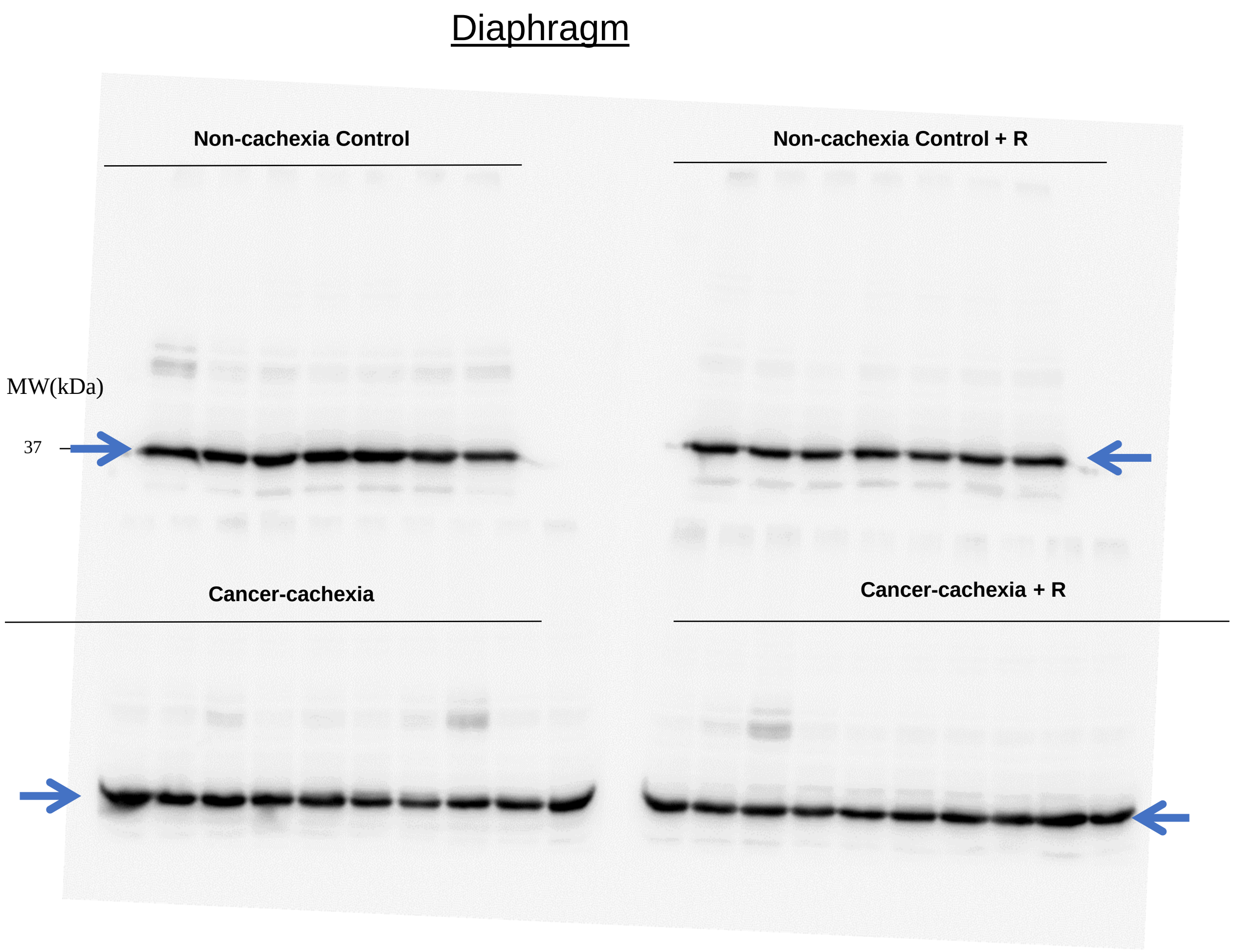

Supplement: Supplementary file 1 [file cancers-14-02894-s001.zip › cancers-1732992-supplementary.pdf]
